# Supplementary material for: An atlas of RNA-dependent proteins in cell division reveals the riboregulation of mitotic protein-protein interactions
Source: Nat Commun. 2025 Mar 8;16:2325. doi: 10.1038/s41467-025-57671-3 (PMC11890761; doi:10.1038/s41467-025-57671-3)
Supplement: Supplementary file 1 — Supplementary Information [file 41467_2025_57671_MOESM1_ESM.pdf]

Supplementary Information

An atlas of RNA-dependent proteins in cell division reveals the riboregulation of mitotic protein-protein interactions

Varshni Rajagopal[1,†], Jeanette Seiler[1,†], Isha Nasa[2,3,†], Simona Cantarella[1], Jana Theiss[1], Franziska Herget[1], Bianca Kaifer[1], Melina Klostermann[4,5], Rainer Will[6], Martin Schneider[7], Dominic Helm[7], Julian König[8,9], Kathi Zarnack[4,5], Sven Diederichs[10,11,*], Arminja N. Kettenbach[2,3,*] and Maïwen Caudron-Herger[1,*]

[1]Research Group "RNA-Protein Complexes & Cell Proliferation", German Cancer Research Center (DKFZ), Heidelberg, Germany

[2]Department of Biochemistry and Cell Biology, Geisel School of Medicine at Dartmouth, Hanover, NH, USA

[3]Norris Cotton Cancer Center, Geisel School of Medicine at Dartmouth, Lebanon, NH, USA

[4]Buchmann Institute for Molecular Life Sciences, Frankfurt, Germany

[5]Department of Bioinformatics, University of Würzburg, Würzburg, Germany

[6]Cellular Tools Core Facility, German Cancer Research Center (DKFZ), Heidelberg, Germany

[7]Proteomics Core Facility, German Cancer Research Center (DKFZ), Heidelberg, Germany

[8]Institute of Molecular Biology (IMB), Mainz, Germany

[9]Theodor Boveri Institute, Biocenter, University of Würzburg, Würzburg, Germany

[10]Division of Cancer Research, Department of Thoracic Surgery, Medical Center - University of Freiburg, Faculty of Medicine, University of Freiburg, Freiburg, Germany

[11]German Cancer Consortium (DKTK), partner site Freiburg, a partnership between DKFZ and University Medical Center Freiburg, Freiburg, Germany

[†]Equal contribution

[*]To whom correspondence should be addressed: SD, s.diederichs@dkfz.de; ANK, Arminja.N.Kettenbach@dartmouth.edu; MCH, m.caudron@dkfz-heidelberg.de

<u>Supplementary information consists in:</u>

Supplementary Figures 1 to 17

Supplementary Note 1: User Guide for R-DeeP 3.0

Supplementary Data 1, provided separately: Results of the cell cycle-related R-DeeP approach in mitosis and interphase

Supplementary Data 2, provided separately: Atlas of RNA-dependent proteins in cell division

Supplementary Data 3, provided separately: Analysis of the AURKA interaction partners

Supplementary Data 4, provided separately: iCLIP2-Seq analysis of KIFC1-interacting RNAs

Supplementary Data 5, provided separately: List of reagents

Supplementary Figs. 1 to 17

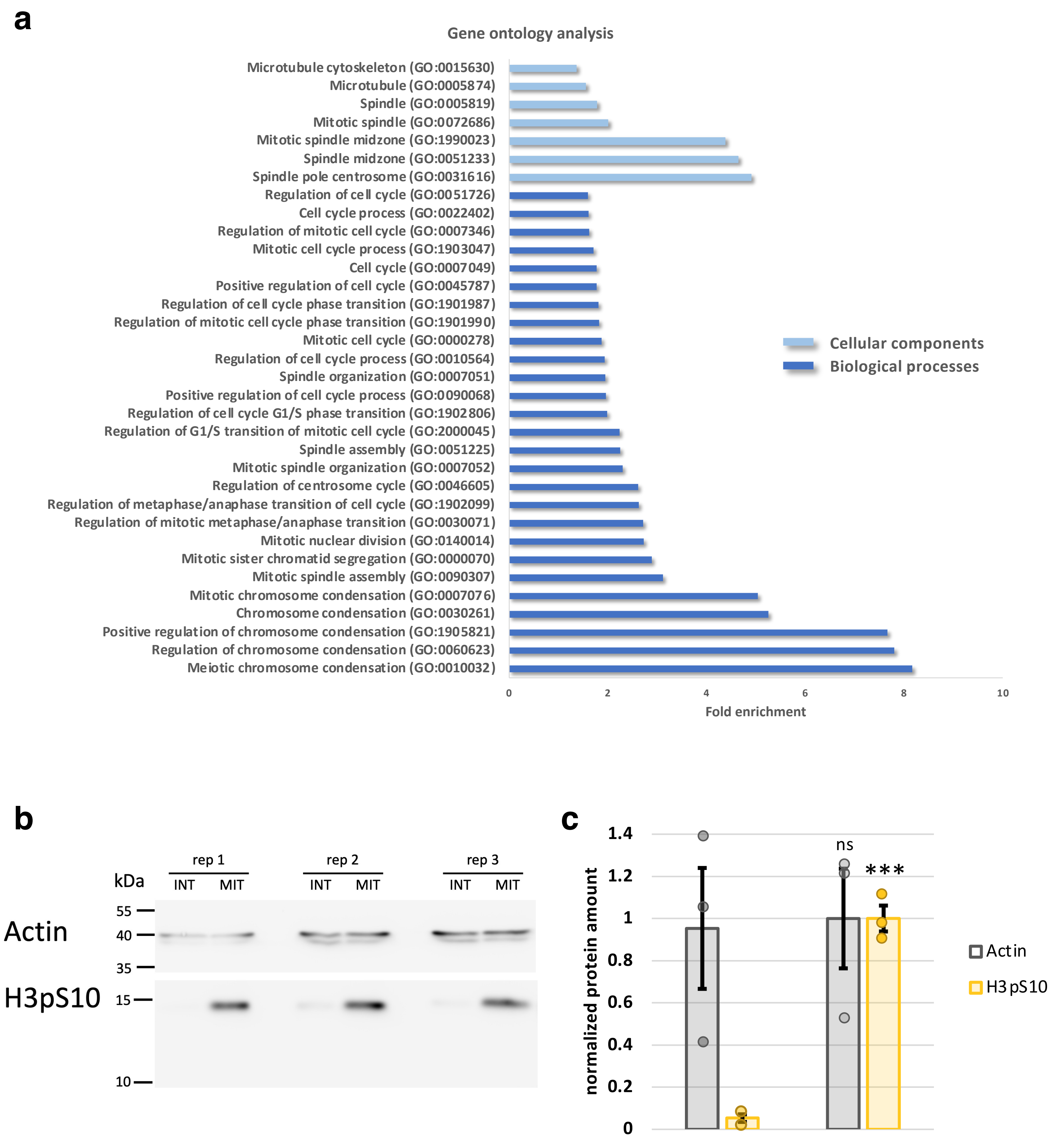

Rajagopal *et al.*, Supplementary Fig. 1

Supplementary Fig. 1. **RNA-dependent proteins are enriched in mitotic factors.**

a A gene ontology (GO) analysis was performed based on the list of RNA-dependent protein from the R-DeeP screen (27). An at least 2-fold significant enrichment was observed for various mitosis-related terms such as cell cycle regulation, spindle and microtubule (adjusted *P*-value < 0.05 according to a Fisher's exact test and FDR correction for multiple testing). Bars in light blue indicate GO analysis for cellular components and bars in dark blue represent GO analysis for biological processes.

b Western blot analysis showing the amount of Histone H3 phosphorylated at Serine 10 (H3pS10) in cell lysate, as indicator of the enrichment in mitotic cells after synchronization in mitosis as compared to interphase. Actin was used as a loading control. Three biological replicates of mitotic and interphasic cell lysates are shown.

c Graph representing quantification of the amount of H3pS10 and actin in the Western blot analysis shown in **b.** The intensity of the bands was quantified using Image J and represented in the bar graph with SEM (N=3). *P*-values were evaluated using a two-tailed, paired t-test (*** *P*-value < 0.001, ns = not significant).

Source data for blots and graphs are provided as Source Data files.

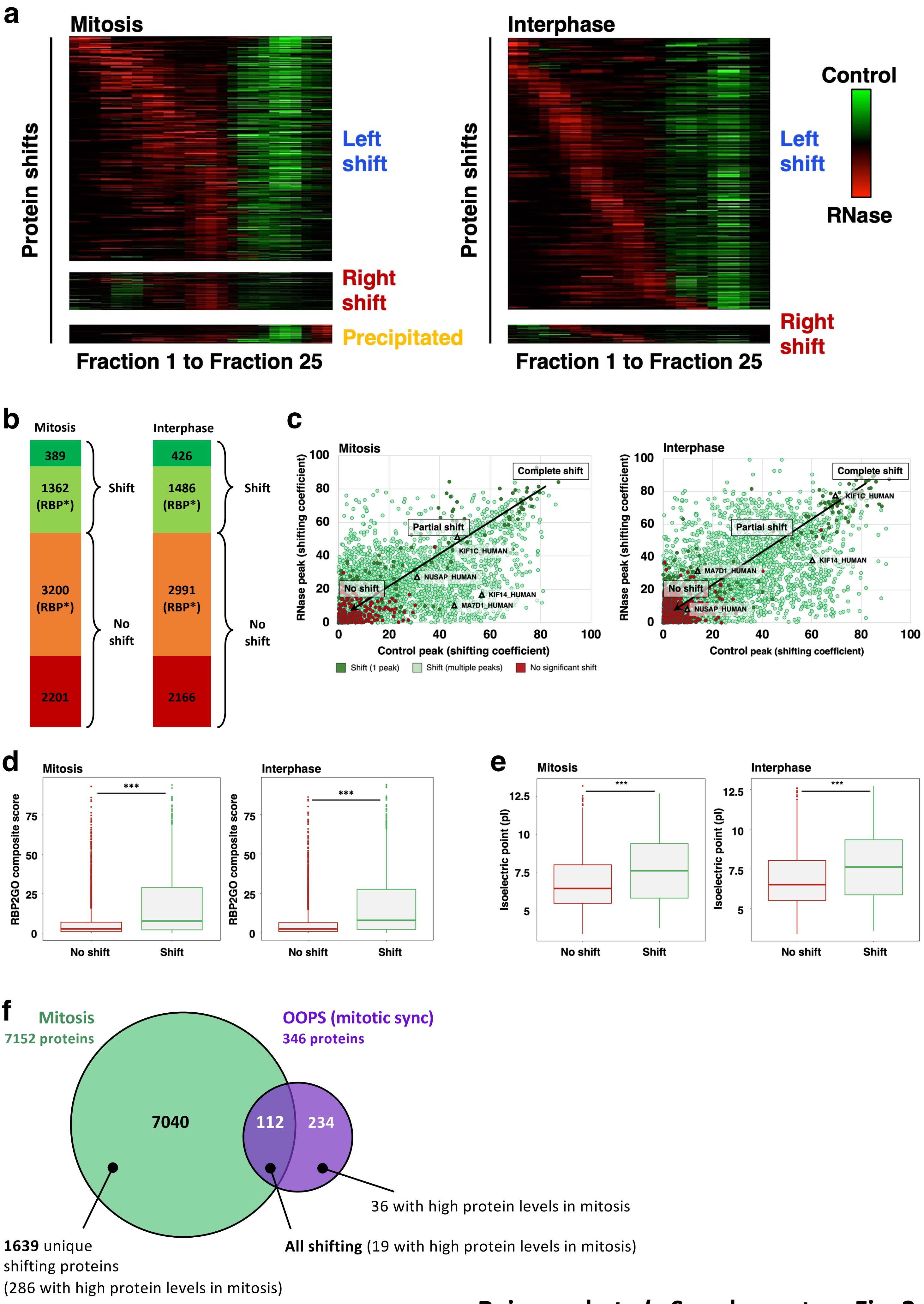

Rajagopal *et al.*, Supplementary Fig. 2

Supplementary Fig. 2. **R-DeeP screens in mitosis and interphase.**

a Heatmaps of the sub-categories for shifting proteins (left shift, right shift, or precipitated), representing the enrichment in the control (green) or in the RNase (red) fractions, for the mitotic and interphasic screen, respectively.

b Classification of the proteins analyzed in the R-DeeP mitotic and interphasic screens according to their shifting behavior and their prior classification in 43 proteome-wide human studies. RNA-dependent proteins not linked to RNA before - in mitosis screen: 389 and interphase screen: 426. RBP*: validated RBPs or RBP candidates.

c Graph depicting the shifting co-efficient of the proteins for each pair of control and RNase peaks. Red: No significant shift, dark green: significant shift from one control peak to one RNase peak, light green: shifts between multiple peaks. For shifts between multiple peaks, they are further categorized as complete shifts: total amount of protein shifting between control and RNase gradients (top right of the graph), partial shift: a of protein shifting between control and RNase gradients (middle) and no shift: no change in shifting pattern (bottom left of the graph). Representative microtubule-related proteins are highlighted on the graph (indicated with their Entry names, see UniProt database).

d Boxplots representing the distribution of the RBP2GO composite score of the proteins classified as non-shifting (No shift) or shifting (Shift). The bar and the box indicate the lower, the median and the upper quartiles. The whiskers represent the range between the bottom of the first quartile and the top of the third quartile, excluding the outliers. The outliers are represented by dots and the *P*-value was calculated using a Wilcoxon test (*** *P*-value < 0.001).

e Same as in **d** for the isoelectric point (pI).

f Venn diagram depicting the intersection (number of detected proteins) between the OOPS study in U2OS cells synchronized in mitosis (OOPS (mitotic sync)) and the new mitotic R-DeeP screen. Protein levels as detected from the analysis of the protein levels in the mitotic R-DeeP screen are indicated below (see also Supplementary Table 1).

Source data for graphs are provided as Source Data files.

a

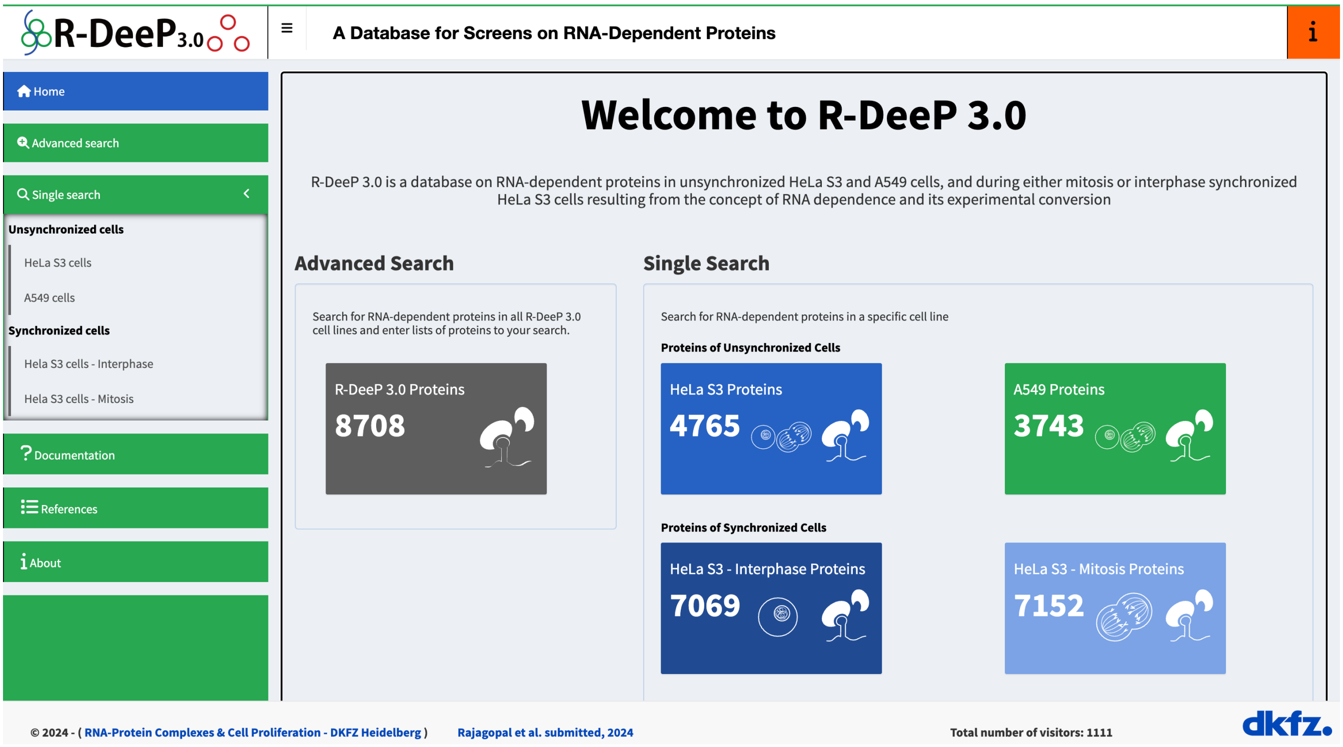

b HeLa S3 cells - synchronized during either Interphase or Mitosis

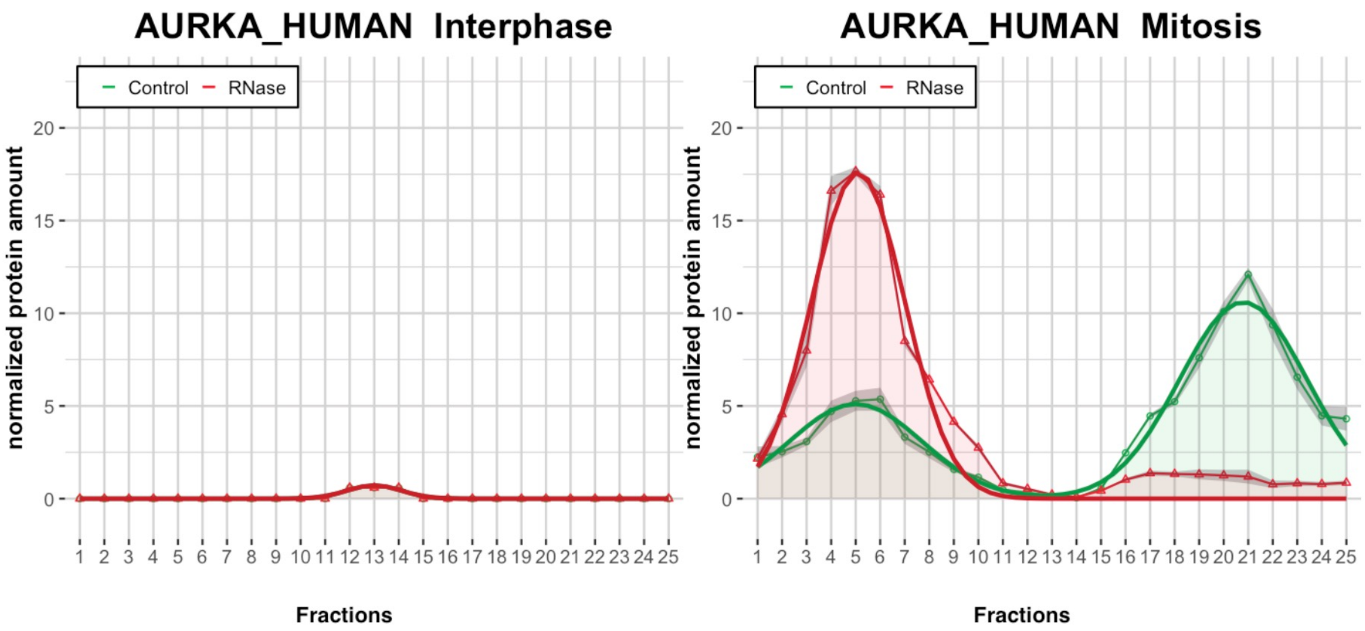

Rajagopal *et al.*, Supplementary Fig. 3

Supplementary Fig. 3. The R-DeeP3 database.

a The R-DeeP 3 database (https://R-DeeP3.dkfz.de) integrates comprehensive information on proteins that are detected in the R-DeeP screens in synchronized HeLa cells at mitosis and interphase, in addition to the knowledge from the two previous screens in unsynchronized HeLa and A549 cells. It provides detailed information on each protein in terms of shift, maxima and offers multiple search and download options. The database is linked to external resources for complementary knowledge on protein structure, sequence and interaction partners.

b Example of comparative R-DeeP representation highlighting the difference in protein amount (here for AURKA) between interphase and mitosis, and the differential shifting properties (here, no shift in interphase, but a clear shift in mitosis). Such a representation is available in R-DeeP3 for all 6059 proteins which were commonly detected in the interphase and mitosis R-DeeP gradients. Left and right panels contain a graphical representation of the protein amount in 25 different fractions of control (green) and RNase-treated (red) sucrose density gradients analyzed by mass spectrometry. Raw data (mean of three replicates) are depicted by line with markers. Smooth lines represent the respective Gaussian fit. The overall protein amount of the raw data was normalized to 100.

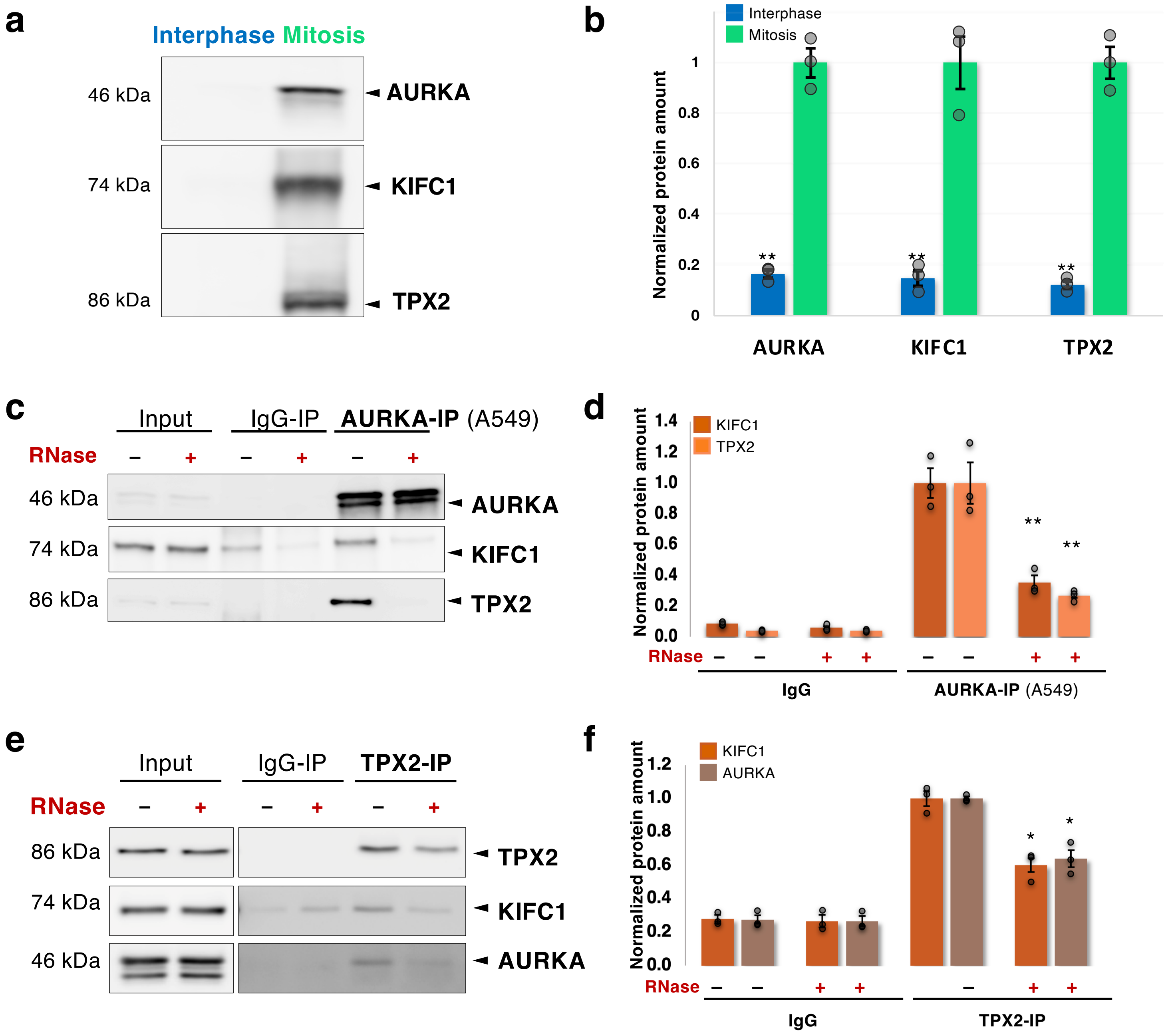

Rajagopal *et al.*, Supplementary Fig. 4

Supplementary Fig. 4. **AURKA interactions with KIFC1 and TPX2 are RNA dependent in mitosis.**

a Representative Western blot analysis (N=3) showing the differential expression of AURKA, KIFC1 and TPX2 in interphasic and mitotic HeLa cell lysates (20 µg total lysate per lane).

b Bar graph representing the total protein amount in interphase and mitotic Hela cell lysates (normalized to mitosis) with SEM (N=3). The *P*-value was calculated using a two-tailed, paired t-test (** *P*-value <0.01).

c Representative Western blot analysis (N=3) showing the immunoprecipitation of AURKA in mitotic A549 cells. AURKA pulldown was performed in the mitotic lysate treated with or without RNase I. Mouse IgG was used as a negative control. KIFC1 (74 kDa) and TPX2 (86 kDa) were pulled down with AURKA (46 kDa) in control samples whereas their interaction was significantly reduced upon RNase treatment (reduction of the band intensity for KIFC1 and TPX2 in the last lane).

d Graph representing the amount of KIFC1 and TPX2 present in IgG and AURKA pulldown samples treated with or without RNase I. The intensities of the Western blot bands were quantified using ImageJ and represented in the bar graph with SEM (N=3). *P*-values were evaluated using a two-tailed, paired t-test (** *P*-value <0.01).

e and **f** same as in **c** and **d**, respectively, for TPX2 pulldown in mitotic HeLa cells synchronized in prometaphase. *P*-value was calculated using two-tailed, paired t-test (* *P*-value <0.05, N=3).

Source data for blots and graphs are provided as Source Data files.

a

PLA TPX2/AURKA (cell cycle phases)

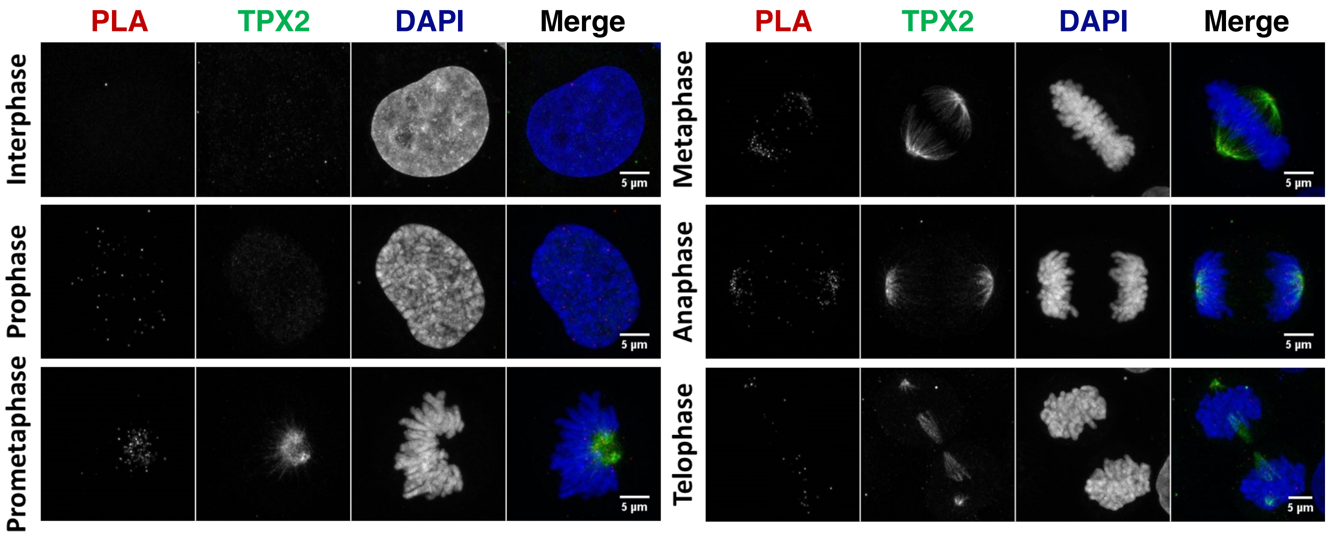

b

PLA TPX2/KIFC1 (cell cycle phases)

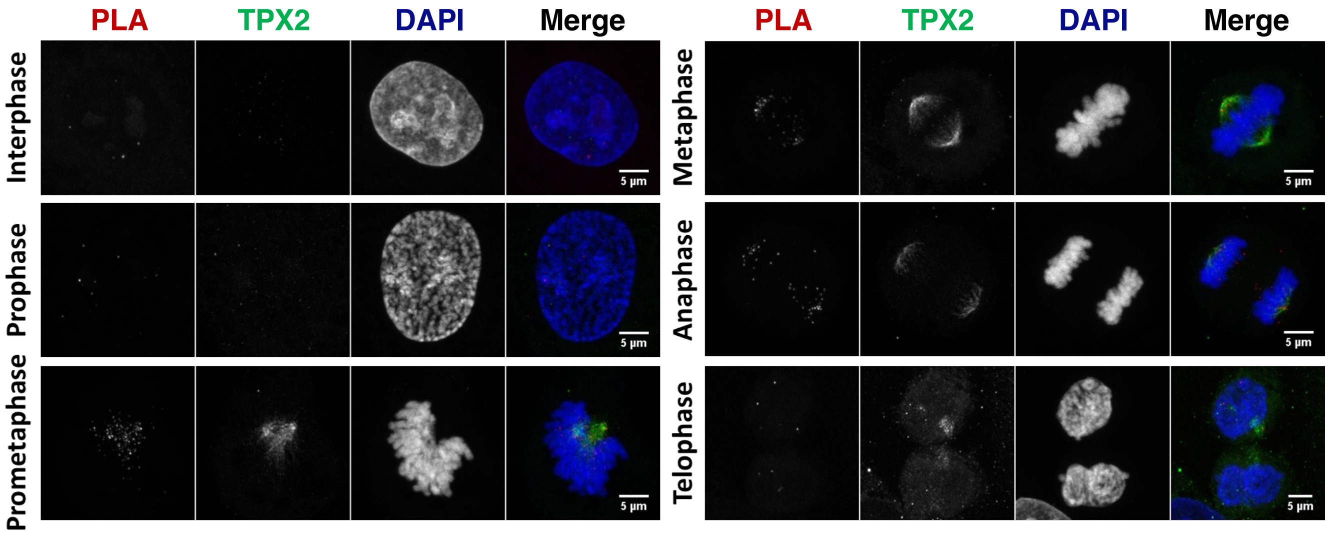

Rajagopal *et al.*, Supplementary Fig. 5

Supplementary Fig. 5. **Protein-protein interactions in mitosis throughout the cell cycle.**

a Representative PLA images (N=3) indicating the close proximity of TPX2 and AURKA in HeLa cells across the cell cycle (interphase to telophase as indicated). The interaction is represented by dots (PLA channel, red dots in the merge channel), TPX2 was stained per immunofluorescence (green) and DNA was stained using DAPI (blue). Scale bar, 5 µm.

b same as in **a** for TPX2 and KIFC1 (N=3, respectively).

Raw images are provided as Source Data files.

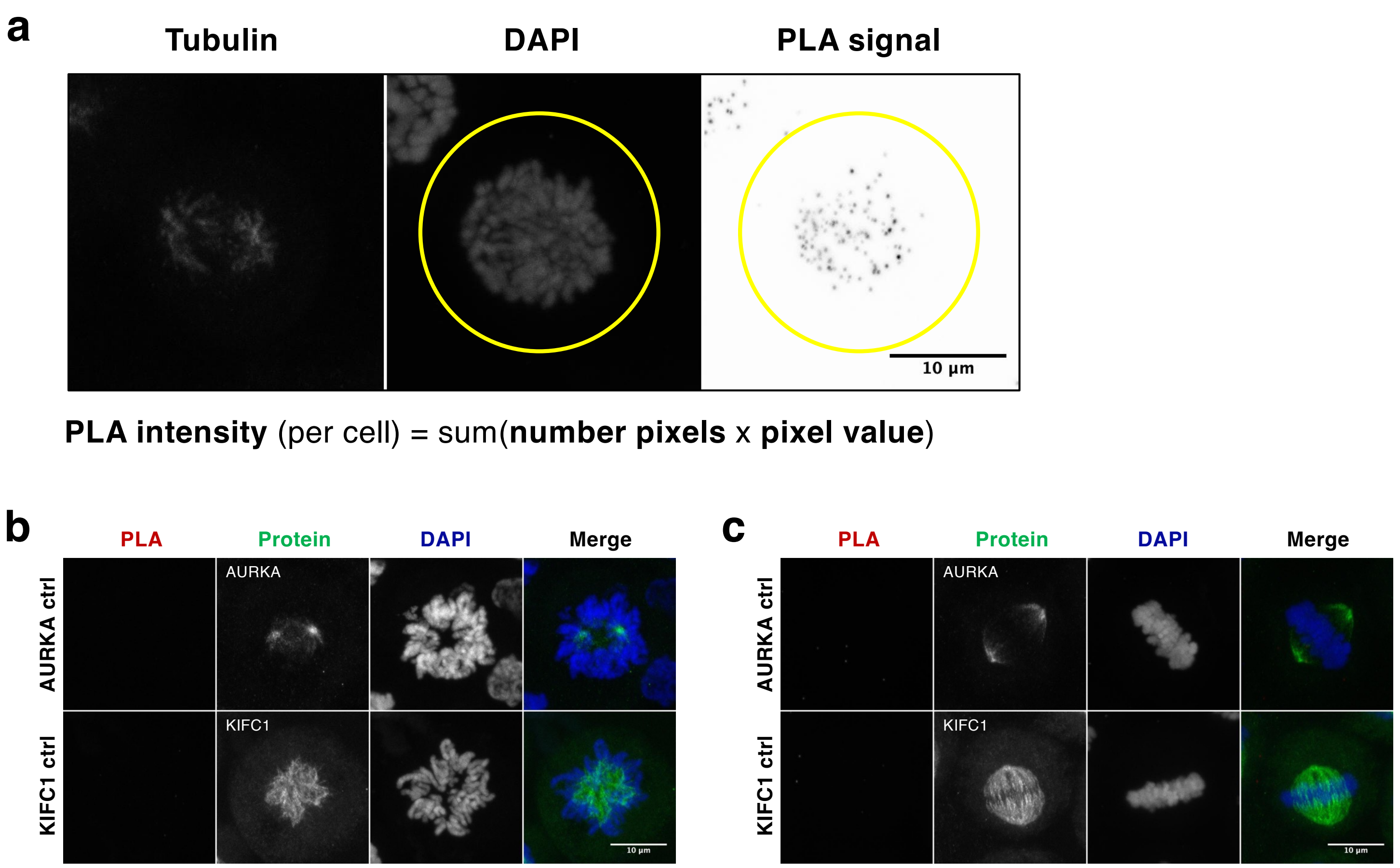

Rajagopal *et al.*, Supplementary Fig. 6

Supplementary Fig. 6. Analysis of the Proximity Ligation Assay (PLA) and associated controls.

a Principle of the analysis of the PLA signal intensity. For each image series, a common circular region of interest (ROI) was placed around the chromosomes. After background signal depletion, the signal intensity (pixel values) of the PLA image was collected and the overall signal intensity inside the ROI was calculated as the sum of each pixel value times the number of pixel with this value. Scale bar, 10 µm.

b Representative PLA images of the PLA assay control samples in HeLa cells synchronized in prometaphase (N=3). The aim of these images is to estimate the amount of background PLA signal generated by the antibody alone and not via the proximity of the two proteins. Here, the cells were probed with only one antibody against AURKA or KIFC1 (in green) and further processed for the PLA assay. It appears, that each antibody alone did not produce any strong PLA signal. Scale bars, 10 µm. Related to Figure 6b and 6c.

c same as in **b**, for HeLa cells synchronized in metaphase (N=3, related to Figure 6d and 6e).

Raw images are provided as Source Data files.

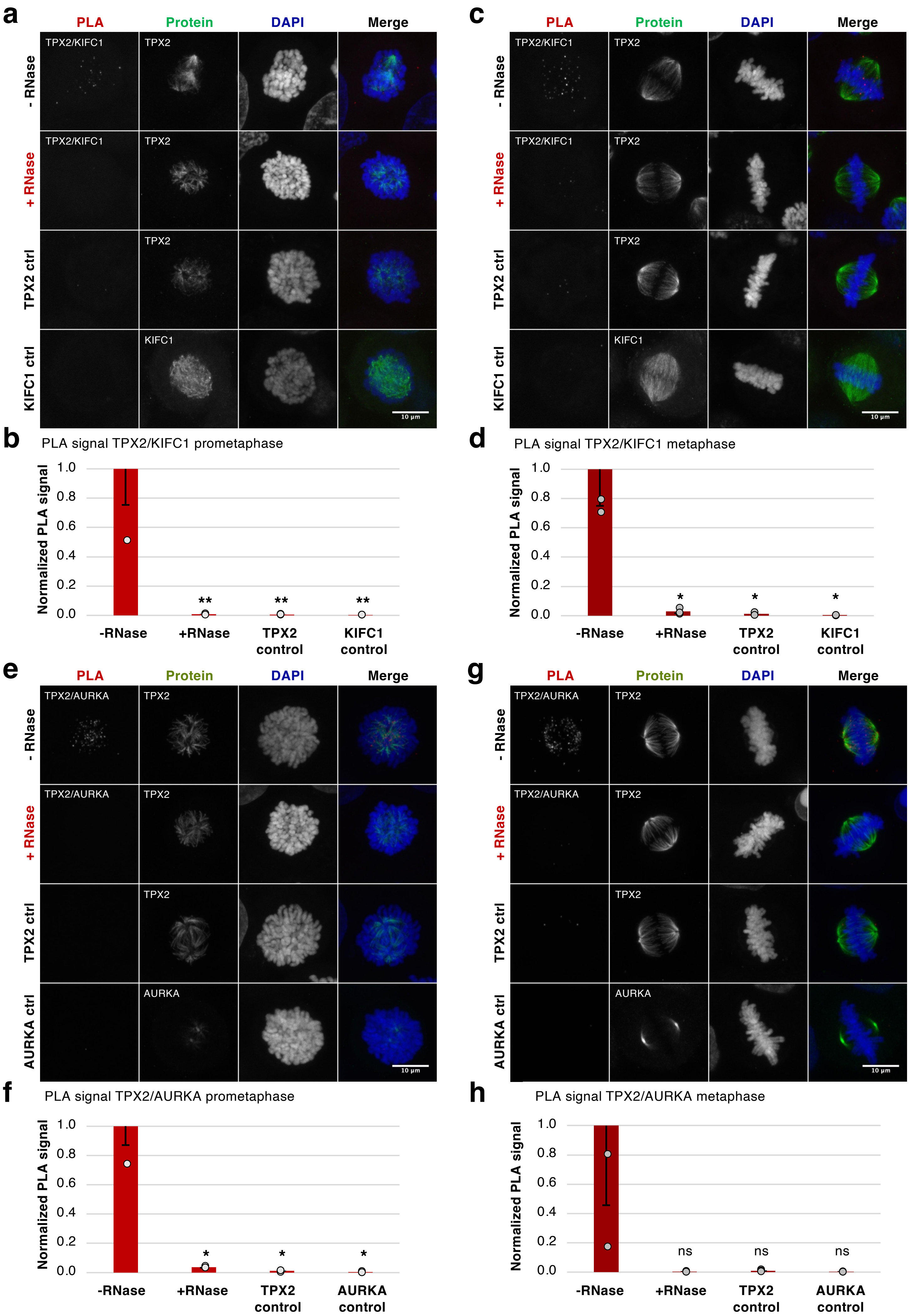

Rajagopal *et al.*, Supplementary Fig. 7

Supplementary Fig. 7. RNA-dependent protein interactions in mitosis demonstrated by Proximity Ligation Assay (PLA).

a PLA images (N=3) showing the proximity of TPX2 and KIFC1 in HeLa cells at prometaphase (PLA channel, red dots in the merge channel). The protein (TPX2 or KIFC1) is seen in green (immunostaining) and DNA was stained using DAPI (blue). The two top images depict representative images in control cells and RNase-treated cells. The two bottom images depict representative images of the individual antibodies PLA controls. Scale bars, 10 µm.

b Bar graph of the quantification of the PLA signal for the proximity of TPX2 and KIFC1 in HeLa prometaphase cells in absence (-RNase) or presence (+RNase) of RNase treatment, as well as in the individual antibody control PLA assays. The signal intensities in each sample were normalized to the signal intensity of the -RNase sample. The error bars indicate the SEM (N=3). The *P*-values were calculated using a two-tailed, unpaired t-test (* *P*-value < 0.05, ** *P*-value <0.01, ns = not significant).

c and **d** same as in **a** and **b** respectively, at metaphase (N=3).

e and **f** same as in **a** and **b**, for TPX2 and AURKA in HeLa cells synchronized in prometaphase (N=3).

g and **h** same as in **a** and **b**, for TPX2 and AURKA in HeLa cells synchronized in metaphase (N=3).

In panels **b**, **d**, **f** and **h**, the upper error bar , which have the same size as the lower error bars, were removed to represent all “-RNase” reference bars with the same height for better comparison.

Source data for graphs and raw images are provided as Source Data files.

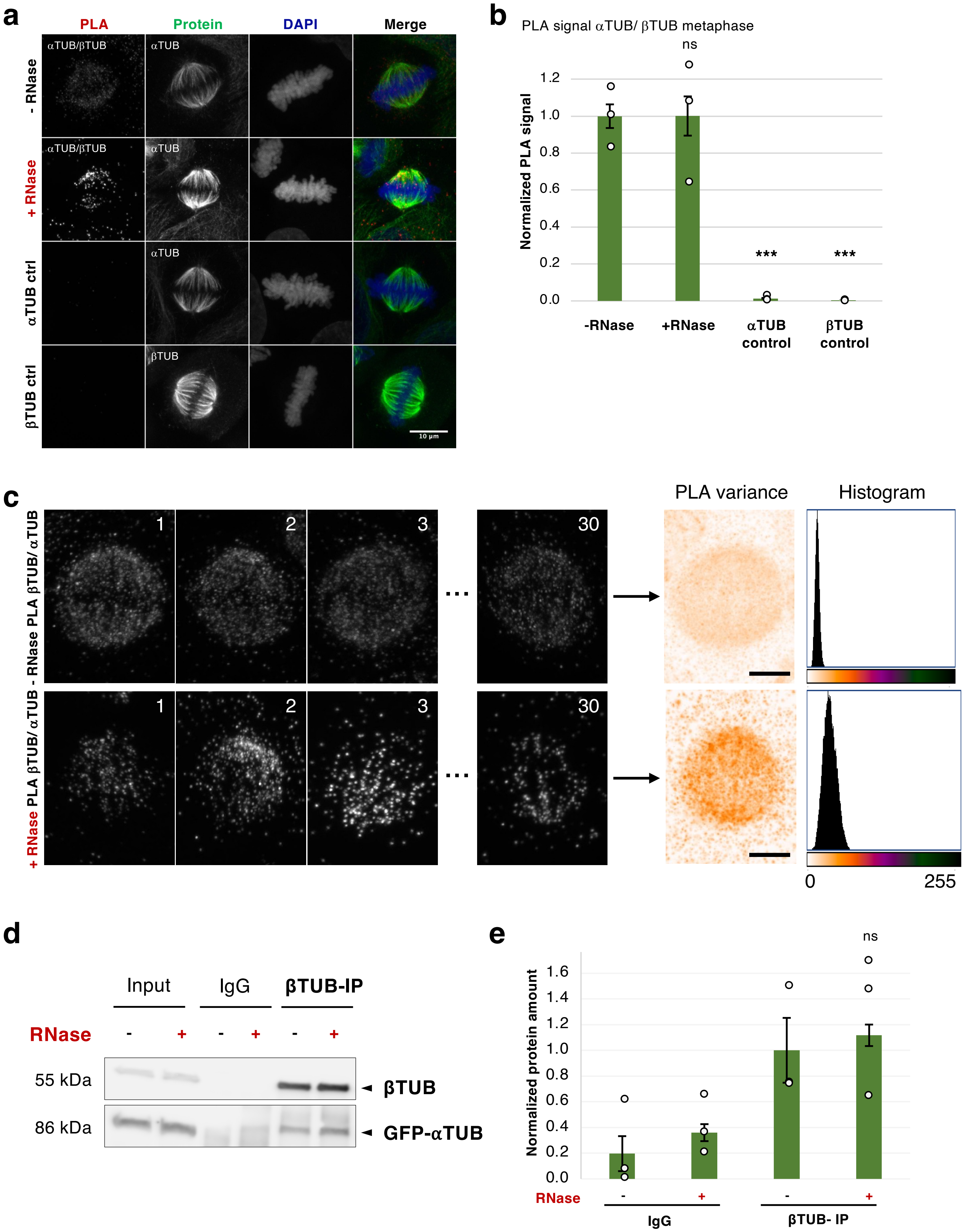

Rajagopal *et al.*, Supplementary Fig. 8

Supplementary Fig. 8. **Interaction between α– and β– tubulin upon RNase treatment.**

a PLA images (N=3) showing the proximity of α– and β– tubulin in HeLa cells synchronized in prometaphase (PLA channel, red dots in the merge channel). The protein (the indicated tubulin) is seen in green (immunostaining) and DNA was stained using DAPI (blue). The two top images depict representative images in control cells and RNase-treated cells. The two bottom images depict representative images of the individual antibodies PLA controls. Scale bars, 10 µm.

b Bar graph of the quantification of the PLA signal for the proximity of α– and β– tubulin in HeLa metaphase cells in absence (-RNase) or presence (+RNase) of RNase treatment, as well as in the individual antibody control PLA assays. The signal intensities in each sample were normalized to the signal intensity of the -RNase sample. The error bars indicate the SEM (N=3). The *P*-values were calculated using a t-test (*** *P*-value < 0.001, ns = not significant). The PLA signal for the proximity of α– and β– tubulin is not significantly decreasing upon RNase treatment, as compared to the untreated (-RNase) sample.

c Differential analysis of the PLA signal for the proximity of α– and β– tubulin. Left panel: images of three replicates (N=3 x 10 images) were rotated, scaled to the average pole-to-pole distance, cropped and superposed to compute an image (orange) representing the variance of the PLA signal in the two series of images (right panel). As seen from the PLA images, the PLA variances and the histograms, the PLA signal for the proximity of α- and β-tubulin after RNase treatment is remarkably inhomogeneously distributed, especially throughout the spindle area (greater variance values). Scale bar, 5 µm.

d Representative Western blot (N=3) analysis of the pulldown of β-tubulin (βTUB) and co-pulldown of α-tubulin tagged with GFP (GFP-αTUB) in HeLa prometaphase cell lysates treated with or without RNase. IgG was used as a negative control. αTUB was detected using an anti-GFP antibody.

e Bar plot showing the quantification of the pulldown of β-tubulin (βTUB) and co-pulldown of α-tubulin tagged with GFP (GFP-αTUB) in HeLa prometaphase cell lysates treated with or without RNase, as in D. The error bars represent the SEM (N=3). The *P*-value was calculated using a two-tailed, unpaired t-test (ns = not significant).

Source data for graphs and raw images are provided as Source Data files.

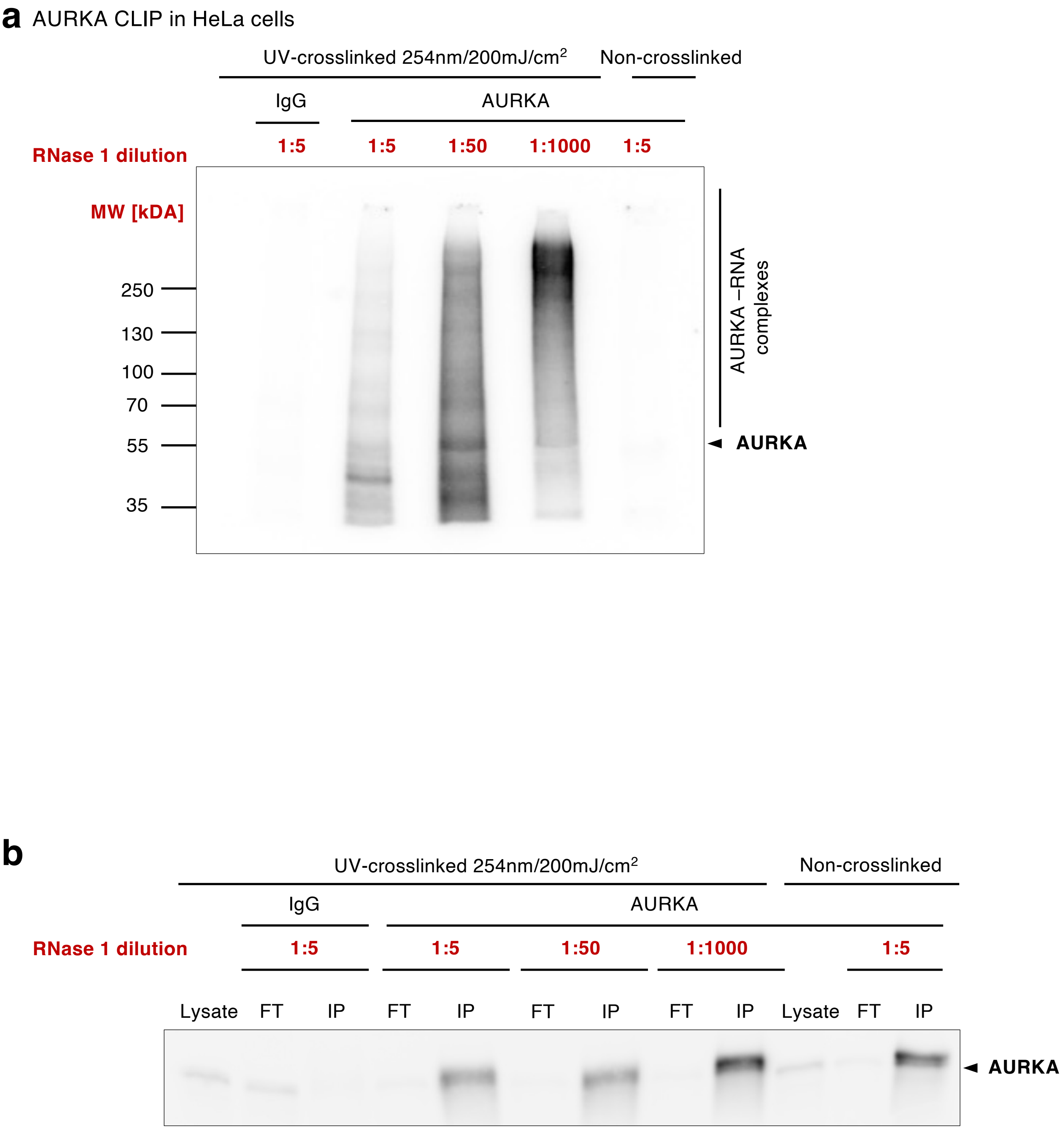

Rajagopal *et al.*, Supplementary Fig. 9

Supplementary Fig. 9. AURKA binds directly to RNA in mitotic HeLa cells.

a Same image as in Figure 7a. Autoradiography indicating the direct binding of AURKA to RNA by iCLIP indicated by shifting of the radioactive RNA signal towards higher molecular weights with decreasing RNase I concentrations in HeLa prometaphase cells. The non-crosslinked sample is used as a control for UV-crosslinking which indicates the absence of RNA signal due to the lack of covalent bond between the protein and RNA (one representative image out of N=3 biological replicates is shown).

b Control IP of AURKA showing the amount of protein in each sample as in **a** (pulldown efficiency, N=3).

Source data for blots are provided as Source Data files.

a AURKA CLIP in HeLa cells using EGFP-AURKA or upon AURKA siRNA

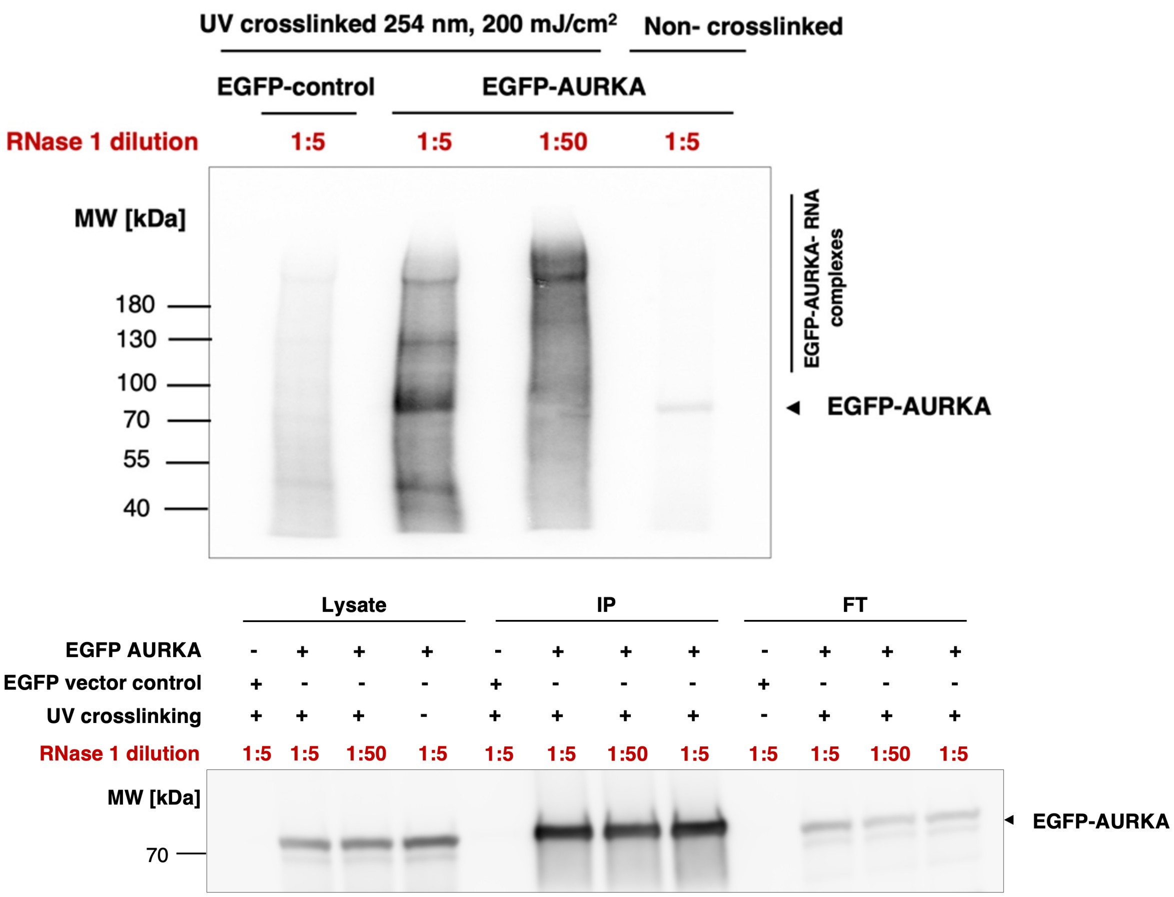

b

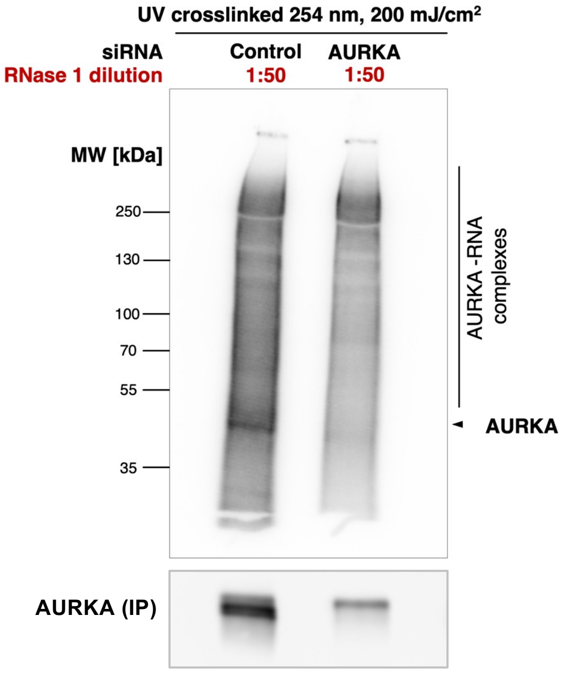

c

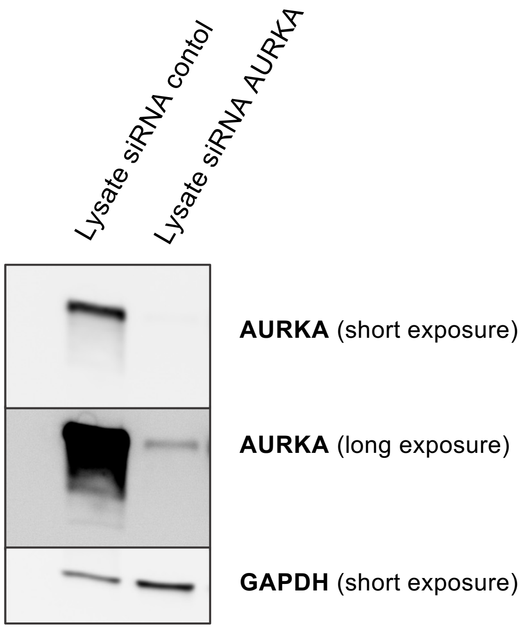

Rajagopal *et al.*, Supplementary Fig. 10

Supplementary Fig. 10. Control CLIP of AURKA in mitotic HeLa cells.

a Top panel: Autoradiography indicating the direct binding of EGFP-AURKA to RNA by iCLIP indicated by a shift of the radioactive RNA signal towards higher molecular weights with decreasing RNase I concentrations in HeLa prometaphase cells. The EGFP-control and non-crosslinked sample are used as controls (one representative image out of N=3 biological replicates is shown). Bottom panel: results of the corresponding IP showing the protein amounts in the input samples (Lysate), IP and flow through (FT).

b Top panel: Autoradiography indicating the direct binding of AURKA to RNA by iCLIP in an siRNA control sample as compared to an AURKA siRNA sample, where the signal is reduced (one representative image out of N=3 biological replicates is shown). Bottom panel: results of the corresponding IP showing the protein amounts in the IP. It is noted that even if AURKA appears to be strongly reduced in the input sample after AURKA siRNA treatment as compared to the control siRNA (as seen in **c**), there is still a residual AURKA signal in the IP after siRNA-mediated knockdown of AURKA (lane 2). One representative image out of N=3 biological replicates is shown.

c Control IP of AURKA showing the amount of protein after siRNA-control and siRNA-mediated knockdown of AURKA. The signal for AURKA in the siRNA lysate (lane 2) is only visible upon long exposure time, resulting in a highly saturated signal in the siRNA AURKA lysate (lane 1). One representative image out of N=3 biological replicates is shown.

Source data for blots are provided as Source Data files.

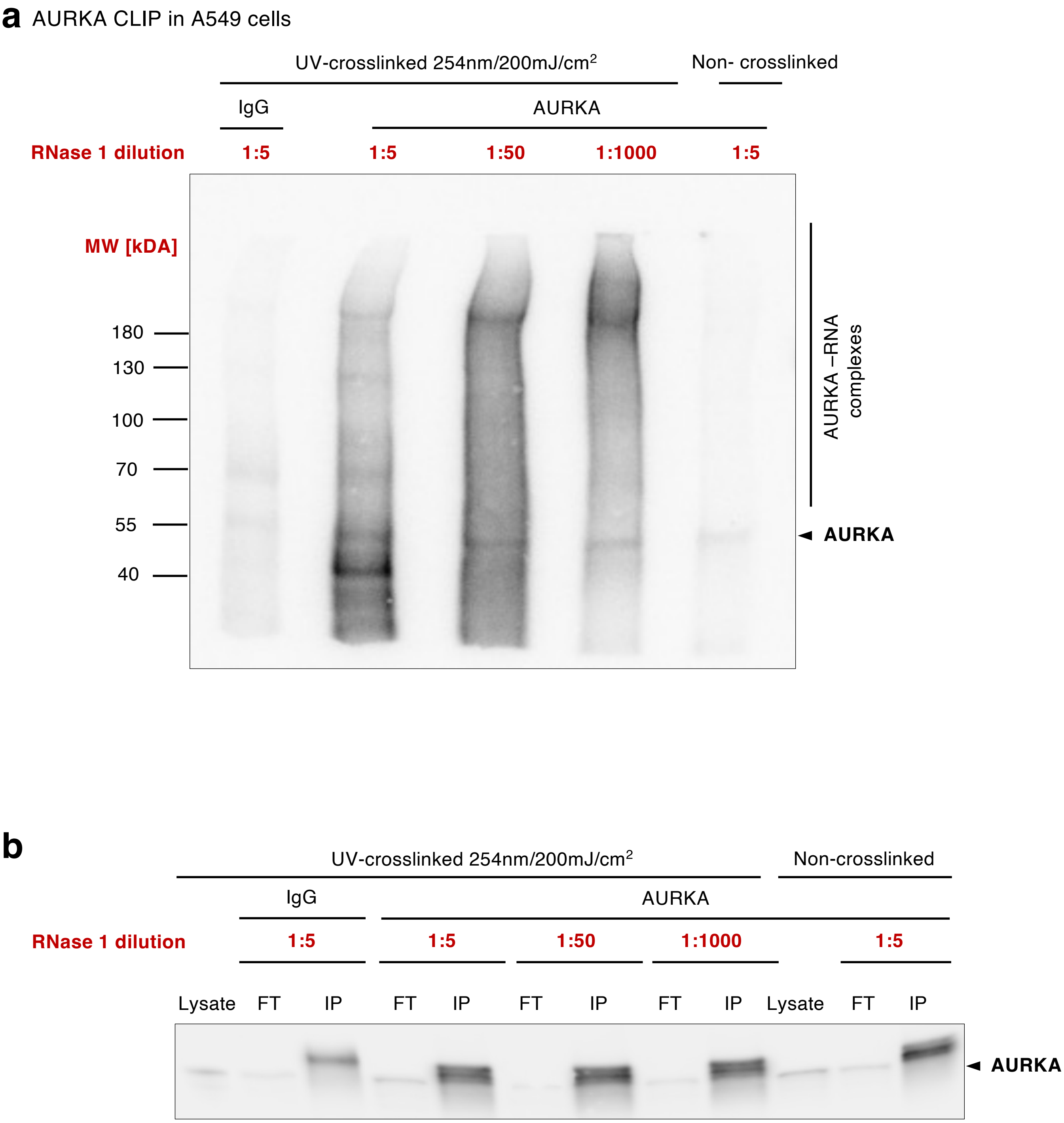

Rajagopal *et al.*, Supplementary Fig. 11

Supplementary Fig. 11. AURKA binds directly to RNA in mitotic A549 cells.

a Autoradiography indicating the direct binding of AURKA to RNA by iCLIP indicated by shifting of the radioactive RNA signal towards higher molecular weights with decreasing RNase I concentrations in A549 prometaphase cells. The non-crosslinked sample is used as a control for UV-crosslinking which indicates the absence of RNA signal due to the lack of covalent bond between the protein and RNA (one representative image out of N=3 biological replicates is shown).

b Control IP of AURKA showing the amount of protein in each sample as in **a** (pulldown efficiency, N=3).

Source data for blots are provided as Source Data files.

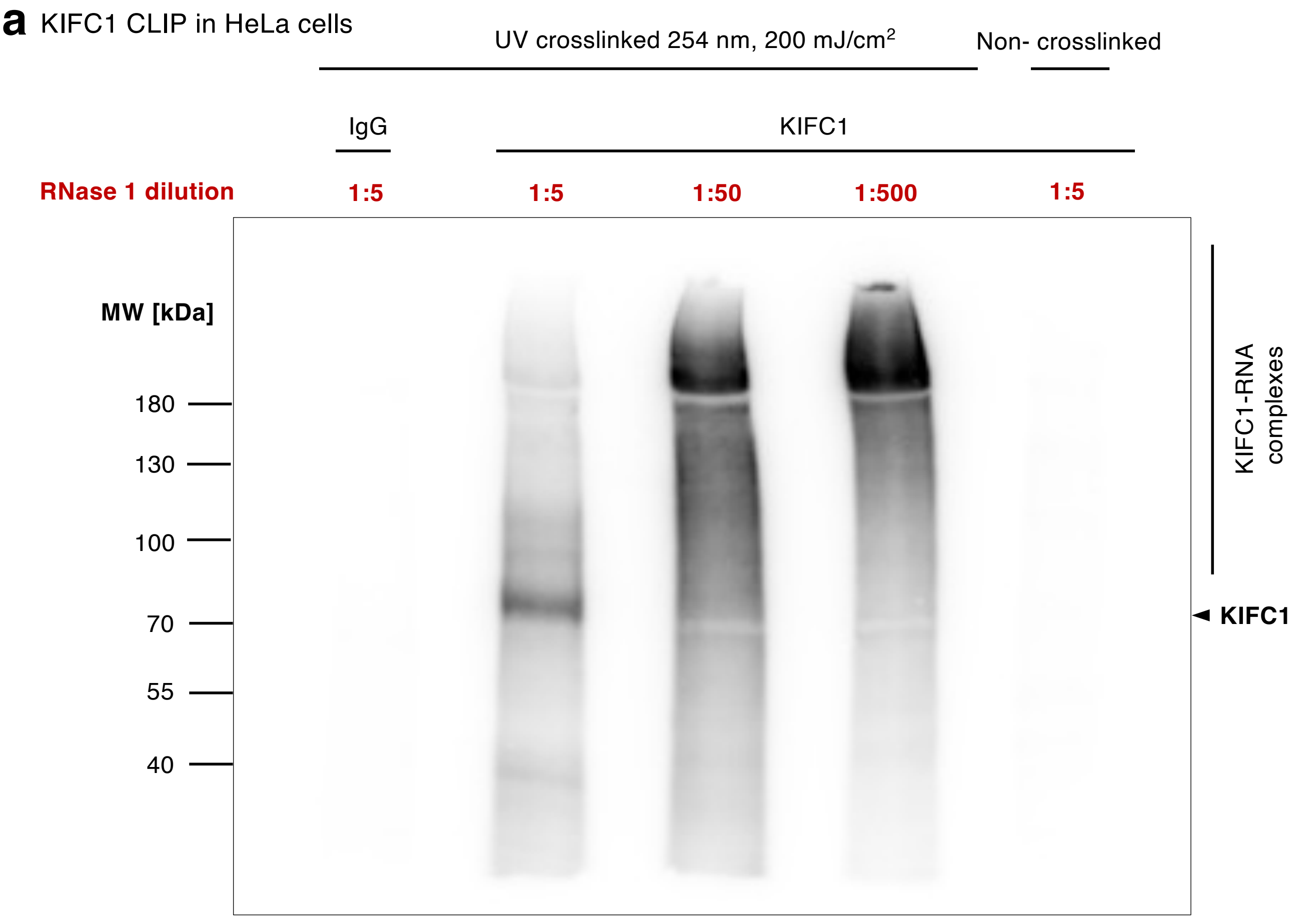
a
KIFC1 CLIP in HeLa cells
UV crosslinked 254 nm, 200 mJ/cm2
Non- crosslinked
IgG
KIFC1
RNase 1 dilution
1:5
1:5
1:50
1:500
1:5
MW [kDa]
180
130
100
70
55
40
KIFC1-RNA complexes
KIFC1

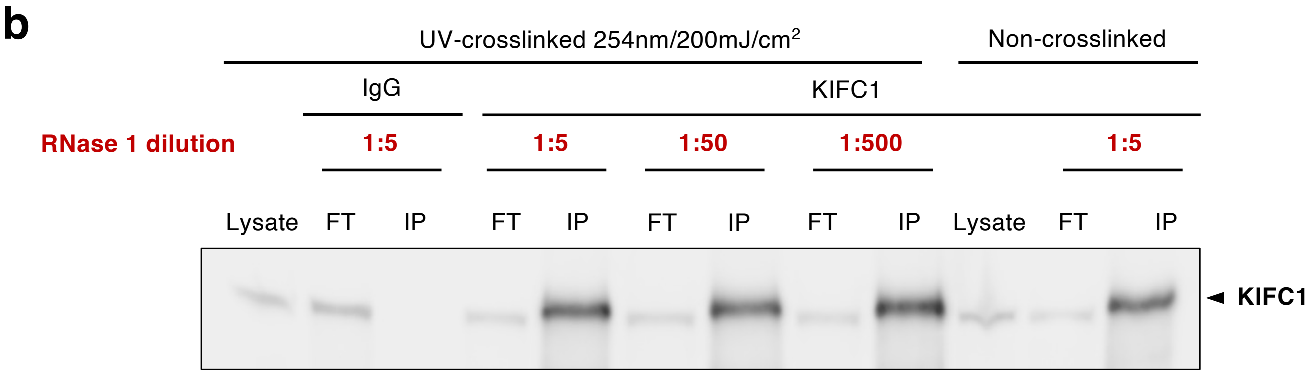
b
UV-crosslinked 254nm/200mJ/cm2
Non-crosslinked
IgG
KIFC1
RNase 1 dilution
1:5
1:5
1:50
1:500
1:5
Lysate
FT
IP
FT
IP
FT
IP
FT
IP
Lysate
FT
IP
KIFC1

Supplementary Fig. 12. KIFC1 binds directly to RNA in mitotic HeLa cells.

a Same image as in Figure 7b. Autoradiography indicating the direct binding of KIFC1 to RNA by iCLIP indicated by shifting of the radioactive RNA signal towards higher molecular weights with decreasing RNase I concentrations in HeLa prometaphase cells. The non-crosslinked sample is used as a control for UV-crosslinking which indicates the absence of RNA signal due to the lack of covalent bond between the protein and RNA (one representative image out of N=3 biological replicates is shown).

b Control IP of KIFC1 showing the amount of protein in each sample as in **a** (pulldown efficiency, N=3).

Source data for blots are provided as Source Data files.

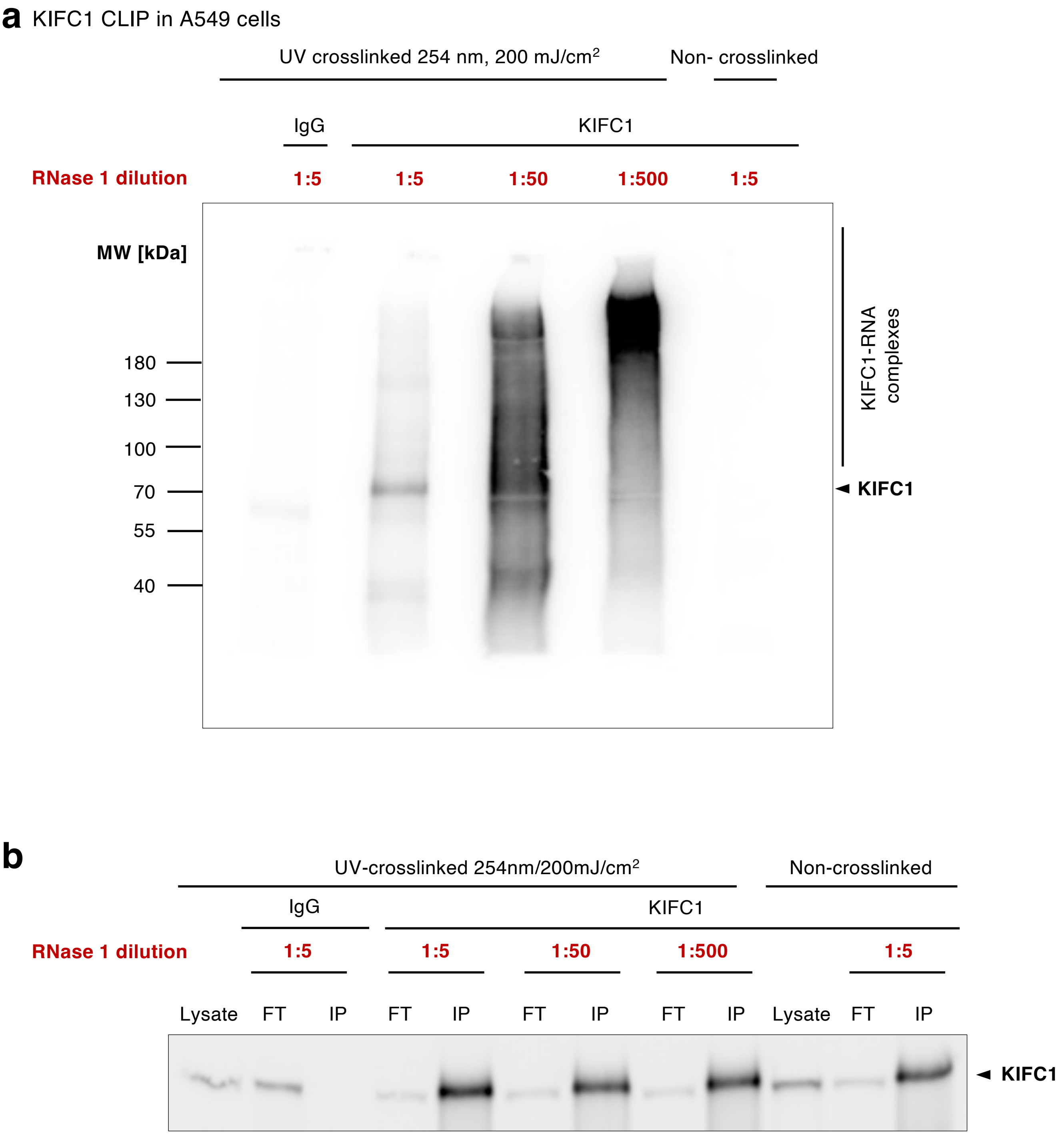

Rajagopal *et al.*, Supplementary Fig. 13

Supplementary Fig. 13. KIFC1 binds directly to RNA in mitotic A549 cells.

a Autoradiography indicating the direct binding of KIFC1 to RNA by iCLIP indicated by shifting of the radioactive RNA signal towards higher molecular weights with decreasing RNase I concentrations in A549 prometaphase cells. The non-crosslinked sample is used as a control for UV-crosslinking which indicates the absence of RNA signal due to the lack of covalent bond between the protein and RNA (one representative image out of N=3 biological replicates is shown).

b Control IP of KIFC1 showing the amount of protein in each sample as in **a** (pulldown efficiency, N=3).

Source data for blots are provided as Source Data files.

a TPX2 CLIP in HeLa cells

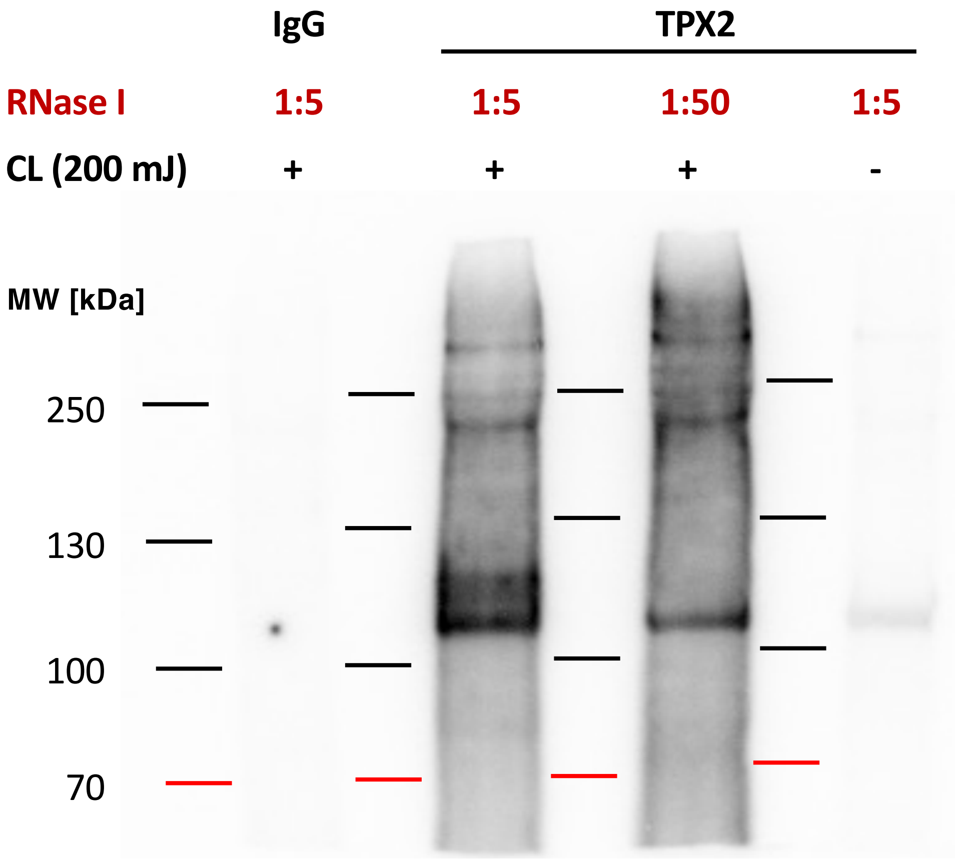

b

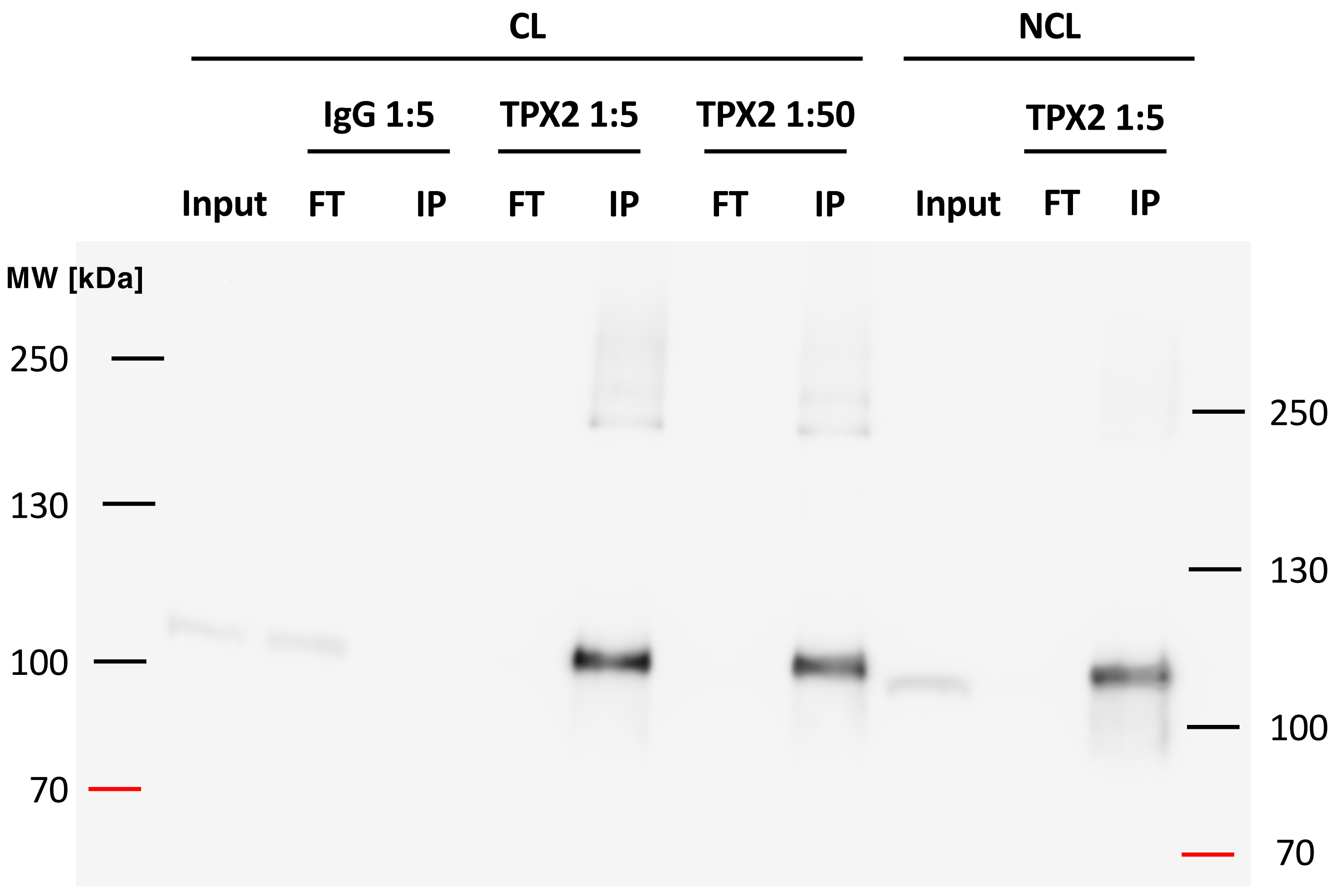

Rajagopal *et al.*, Supplementary Fig. 14

Supplementary Fig. 14. TPX2 binds directly to RNA in mitotic HeLa cells.

a Autoradiography indicating the direct binding of TPX2 to RNA by iCLIP indicated by shifting of the radioactive RNA signal towards higher molecular weights with decreasing RNase I concentrations in HeLa metaphase cells. The non-crosslinked sample is used as a control for UV-crosslinking which indicates the absence of RNA signal due to the lack of covalent bond between the protein and RNA (one representative image out of N=3 biological replicates is shown).

b Control IP of TPX2 showing the amount of protein in each sample as in **a** (pulldown efficiency, N=3).

Source data for blots are provided as Source Data files.

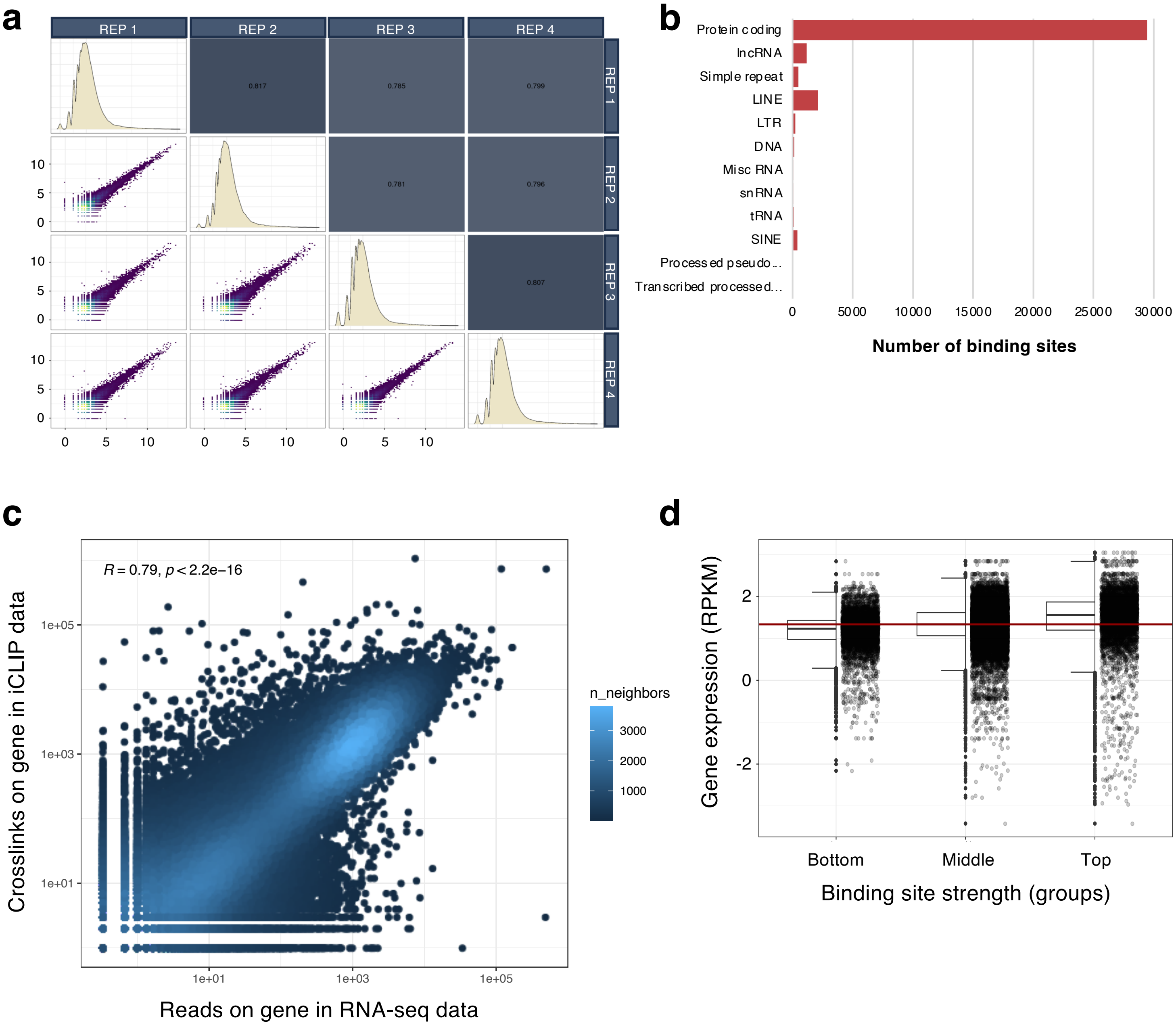

Rajagopal *et al.*, Supplementary Fig. 15

Supplementary Fig. 15. **KIFC1 preferentially binds to protein coding RNAs in prometaphase.**

a Composite graph representing the pairwise correlations between the KIFC1 iCLIP-Seq replicates as scatter plot (bottom left), the Pearson correlation coefficient (upper right) and the coverage distribution as density (diagonal).

b Horizontal bar plot indicating the number of binding sites within the various categories of non-rRNA target genes from the KIFC1 iCLIP-Seq in HeLa prometaphase cells. The genes are classified as protein coding, lncRNA, simple repeat, LINE, LTR, DNA, Misc RNA, snRNA, tRNA, SINE, processed pseudogene and transcribed processed pseudogene.

c Scatter plot showing a significant positive correlation between the number of reads on genes in the RNA-seq data and the number of crosslinks on genes in the iCLIP data (mean of three replicates, Pearson's correlation test).

d Boxplot showing the expression of genes with (bottom 20% of the binding sites), middle or top (top 20% of the binding sites) KIF1C binding site strength (stratified by the apparent KIF1C binding strength according to the PureCLIP score). The red line indicates the median expression of the genes in the middle group to better visualise the difference in median of the three groups.

Code for these analyses is available as Source Data file.

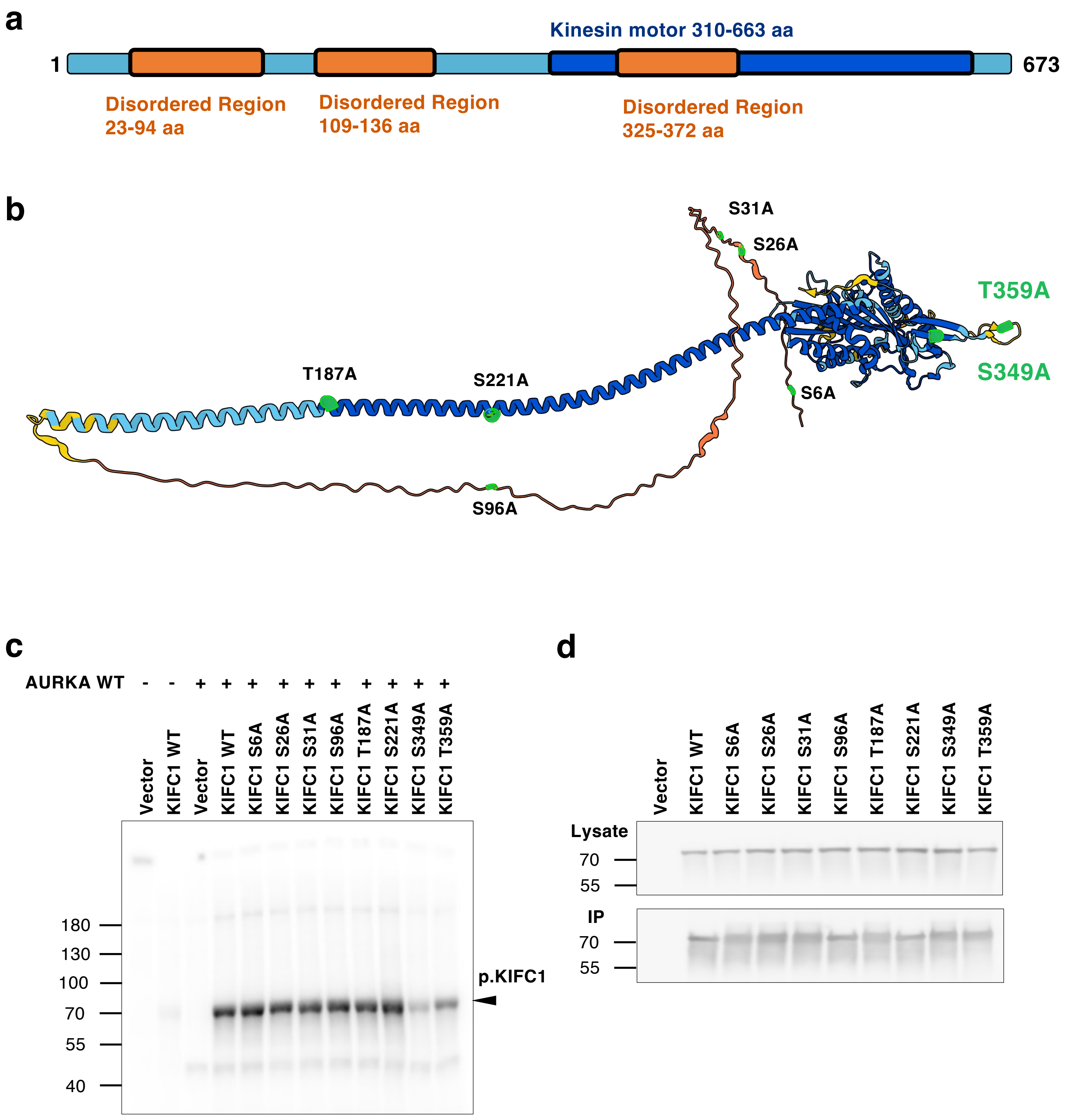

Rajagopal *et al.*, Supplementary Fig. 16

Supplementary Fig. 16. AURKA phosphorylates KIFC1 at S349 and T359

a Schematic representation of KIFC1 indicating the location of the kinase domain and the position of disordered regions across the KIFC1 structure.

b Schematic representation of KIFC1 indicating the position of eight potential phosphorylation sites (highlighted in green) that were identified based on the consensus sequence of AURKA (27). The schematic was generated using AlphaFold (alphafold.ebi.ac.uk).

c Representative autoradiography indicating the phosphorylation intensity of KIFC1 in wild-type (WT) and non-phosphorylatable KIFC1 mutants in the presence of purified AURKA WT (N=3). The *in vitro* kinase assay was performed using KIFC1 pulled down from HeLa prometaphase lysates overexpressing the WT or mutant KIFC1 proteins with an N-terminal Flag-HA tag. An empty vector was used as a negative control. The single mutations of the residues are indicated above each lane.

d Representative Western blot analysis of the expression and pulldown efficiency of Flag-HA-tagged KIFC1 WT and mutants (as in **c**) using anti-DYKDDDDK magnetic agarose beads for *in vitro* kinase assay in HeLa cell lysates (N=3).

Source data for blots are provided as Source Data files.

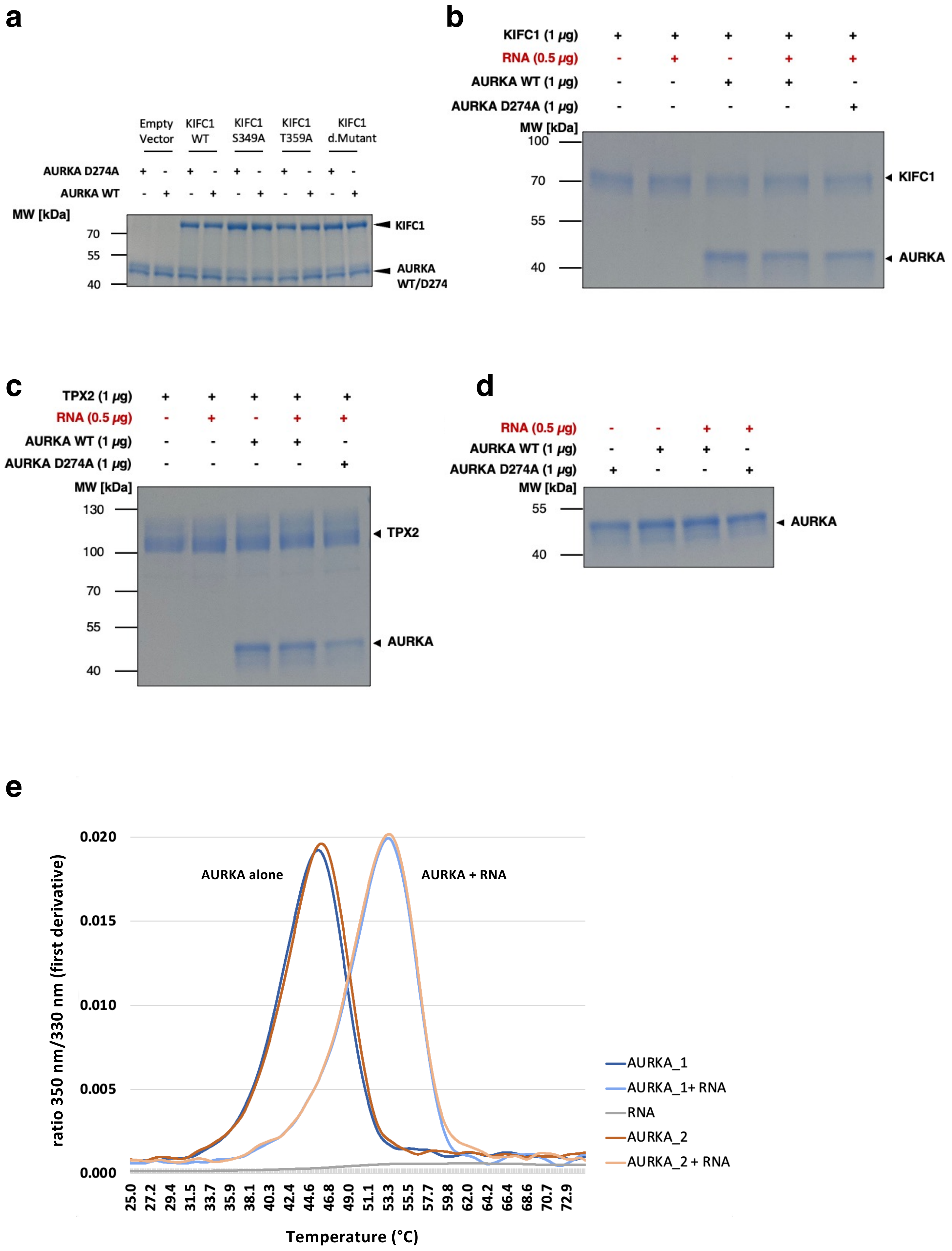

Rajagopal *et al.*, Supplementary Fig. 17

Supplementary Fig. 17. The kinase activity of AURKA is RNA dependent (loading controls).

a Coomassie staining of the proteins (as indicated) added into *the in vitro* AURKA kinase assay as shown in Figure 8a (one representative image out of three replicates is shown).

b Coomassie staining of the proteins (as indicated) added into *the in vitro* AURKA kinase assay as shown in Figure 8c (one representative image out of three replicates is shown).

c Coomassie staining of the proteins (as indicated) added into *the in vitro* AURKA kinase assay as shown in Figure 8d (one representative image out of three replicates is shown).

d Coomassie staining of the proteins (as indicated) added into *the in vitro* AURKA kinase assay as shown in Figure 8e (one representative image out of three replicates is shown).

e Protein unfolding upon exposition to an increasing temperature gradient was monitored by analyzing the fluorescence emission intensities at 350 nm and 330 nm (the first derivative of the ratio F350/F330 is shown) resulting from intrinsic protein fluorescence (tryptophan and tyrosine residues) after excitation at 280 nm. The graph shows the stabilization of AURKA upon addition of total RNA as indicated by a shift towards higher temperatures of AURKA melting temperature as indicated by the inflection point of the curve (compare dark blue or orange curves with light blue or orange curves), in low salt condition (10 mM NaCl). AURKA from two different protein purification preparations was tested (AURKA_1 in blue and AURKA_2 in orange). No changes in ratio were observed with RNA alone in low-salt conditions (grey curve).

Source data for blots and graphs are provided as Source Data files.

Supplementary Note 1

User Guide

R-DeeP 3.0 - Database for RNA-Dependent Proteins (https://R-DeeP3.dkfz.de)

R-DeeP 3.0 is a database for RNA-dependent proteins based on the concept of "RNA dependence" and its direct experimental application.

A protein is defined as "RNA-dependent" if its interactome depends on RNA without necessarily directly binding to RNA as described previously [1][2]. Briefly, RNA-dependent proteins and complexes migrate to different positions in a sucrose density gradient in the control versus the RNase-treated lysates, according to their apparent molecular weight (i.e., according to the size of the complex of which they are part of). This allows the specific and quantitative discovery of RNA-dependent proteins [1][3]. R-DeeP 3.0 is the result of the statistical analysis of proteome-wide mass spectrometry data to determine the behavior of proteins in a sucrose density gradient in presence of RNA molecules and after RNase treatment analyzed in human unsynchronized HeLa S3 and A549 cells (previous datasets) and HeLa S3 cells synchronized in mitosis or interphase (new datasets).

R-DeeP 3.0 provides various search and download options for in-depth analyses, which are further described in this User Guide (**Illustration 1**). In addition, R-DeeP 3.0 offers a summary of multiple RNA-binding protein resources with access to the corresponding publication webpage.

The database R-DeeP 3.0 has been optimized for use with Firefox or Safari browsers.

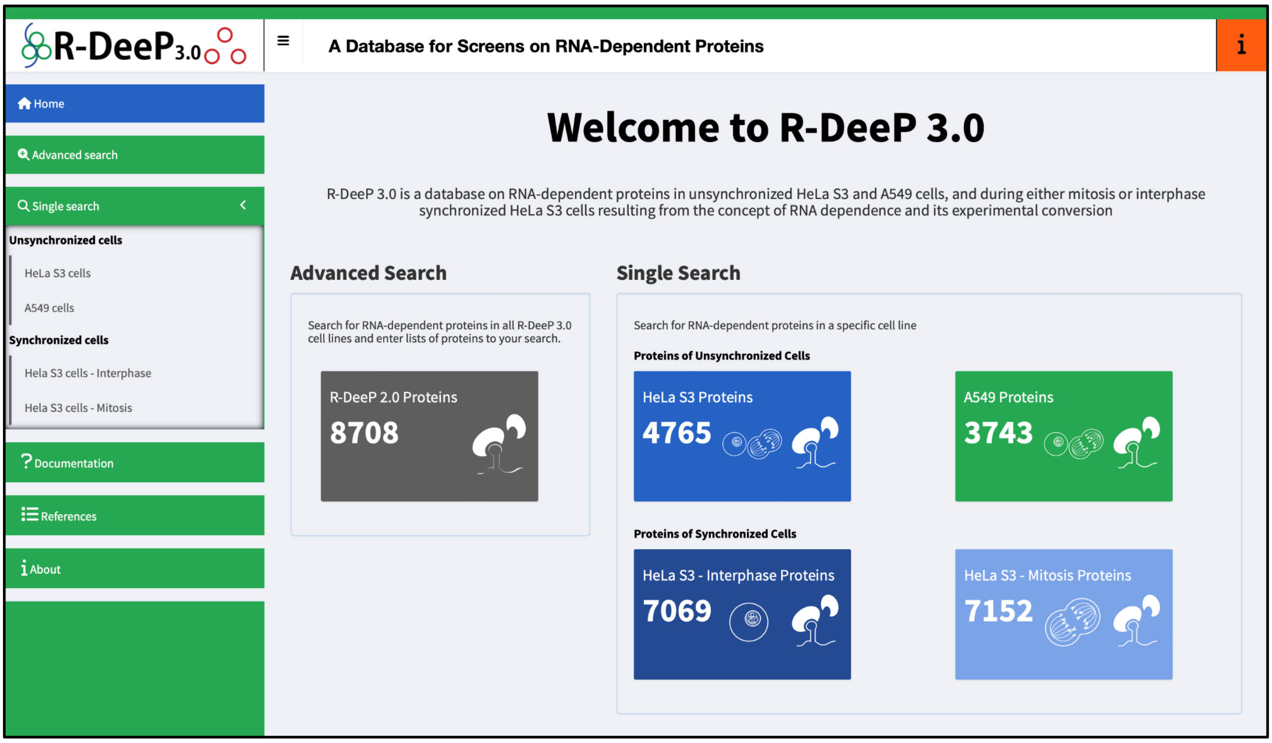

Illustration 1. R-DeeP 3.0 Homepage at https://r-deep3.dkfz.de/. The info boxes for all datasets are clickable and bring the user directly to the Advanced Search or respective Single Search option for each dataset and cell line (see below).

1. Advanced Search

The Advanced search option allows comparing a protein directly between the four datasets. In addition, it provides support for a list of proteins as an input format. Such a list can be copied and pasted into the search field (**Illustration 2**).

The protein names, gene names, Uniprot IDs or other aliases such as accession numbers from HGNC and MIM databases can be entered into the search field and the search is started when the “Enter” key or the “Submit” button is pressed (**Illustration 3**). As soon as the “Submit” button or “Enter” key is pressed, the sidebar is closed to provide more space to display the results. Pressing on the “Sidebar” button will re-open the sidebar. Alternatively, the sidebar can be activated using the small icon right from the R-DeeP 3.0 image at the top left side of the page.

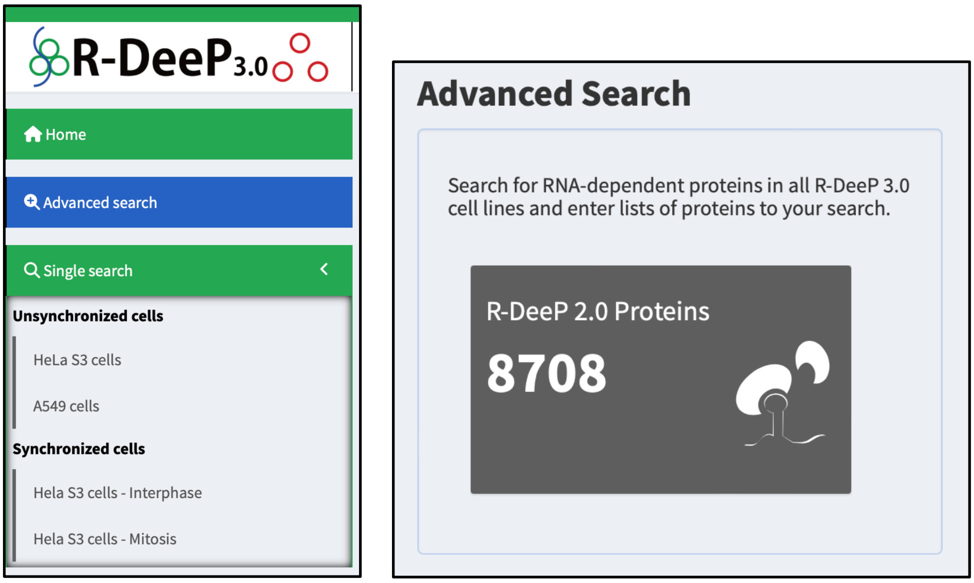

Illustration 2. Advanced search selection from the sidebar (left) or from the info box (right).

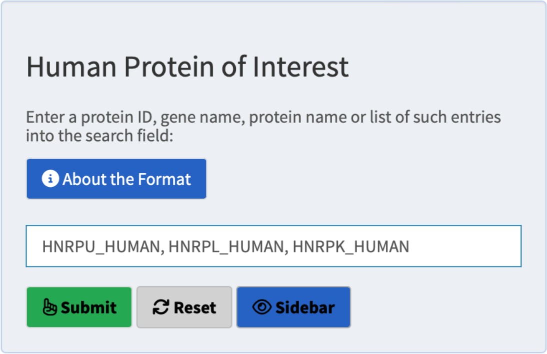

Illustration 3. Advanced search input field.

In the “About the Format” pop-up window, more detailed information about the input format is provided (**Illustration 4**). The pop-up window contains a link to the UniProt database, that could be helpful to find the proper protein or gene name in case they are not known. It also provides more information about the format requirements of a protein list. Besides the regular option to search for a protein or gene name, it is possible to copy and paste a list of proteins from a table. The format of the list needs to strictly adhere to the formatting instructions mentioned in the “About the format” panel (**Illustration 4**).

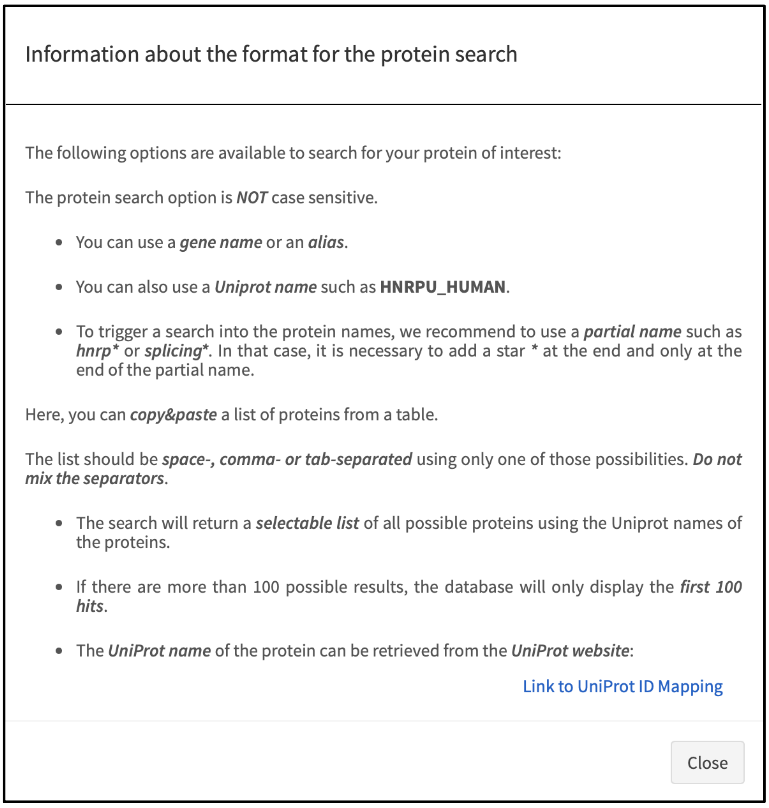

Illustration 4. Information about the search format.

In case of a successful search, a new panel with search results appears with either a unique result (**Illustration 5**) or a list of suggested hits (**Illustration 6**). The search result already indicates whether data is available for this protein and whether the protein is shifting (green comment next to the protein name, clickable button), whether there is data available but the protein does not shift (yellow comment, clickable button) or whether there is no data available, *i.e.* the protein has not been detected by mass spectrometry (red comment, no clickable button, **Illustration 7**). The protein of interest, if available, can be selected to obtain the corresponding comprehensive R-DeeP 3.0 analysis.

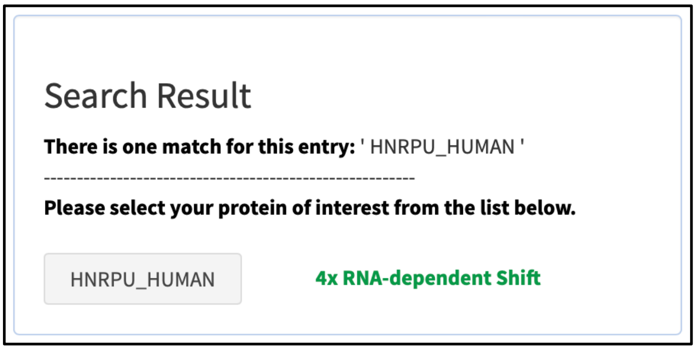

Illustration 5. Advanced search for a single protein.

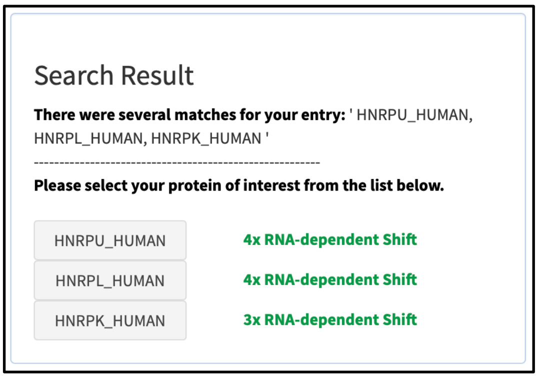

Illustration 6. Advanced search for a list of proteins.

In addition, if the search result displays a list of proteins, it directly indicates whether data is available for the protein and whether it is shifting. However, since the search occurs in all datasets at one time, there are multiple options (**Illustration 7**):

- **4x, 3x or 2x RNA-dependent Shift**: RNA-dependent shift in 4, or in at least 3 or 2 datasets (green comment next to the protein name, clickable button).
- **Shift HeLa**: RNA-dependent shift in HeLa S3 unsynchronized dataset. Similarly, there are proteins with "**Shift A549**", "**Shift HeLa Mitosis**" and "**Shift HeLa Interphase**" (green comment next to the protein name, clickable button). In that case, there is a shift observed in only one of the datasets. For the other datasets, it can be "data available" or "no data" (see below).
- **4x, 3x or 2x Data**: no RNA-dependent shift in 4, 3 or 2 datasets. There is no data available for the other datasets (yellow comment, clickable button).
- **Data HeLa Mitosis**: no RNA-dependent shift in HeLa S3 synchronized in mitosis (yellow comment, clickable button) and no data in any of the other experiment, *i.e.* not detected. Similarly, there are proteins with "**Data HeLa**", "**Data A549**" and "**Data HeLa Interphase**" (yellow comment, clickable button).
- **No data**: no data available for this protein in either dataset (red comment, no clickable button (**Illustration 7**).

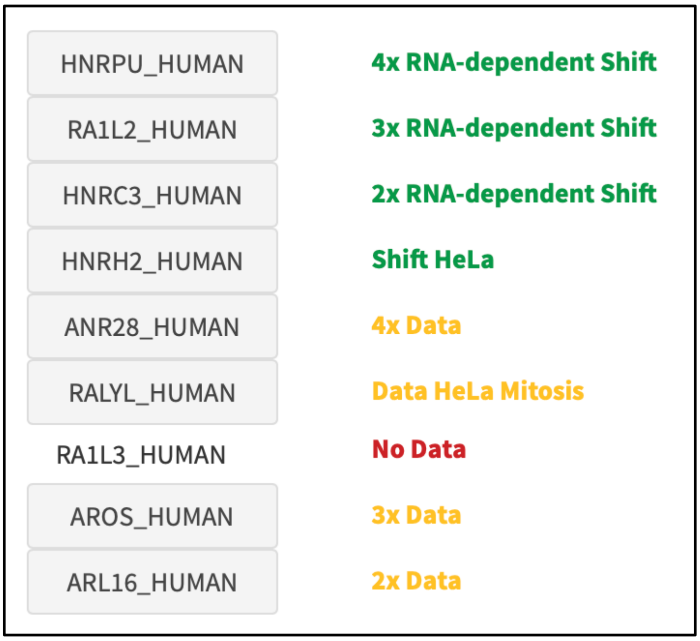

Illustration 7. Example of search results in the Advanced search option.

The search returns an error message in case there are no results (**Illustration 8**).

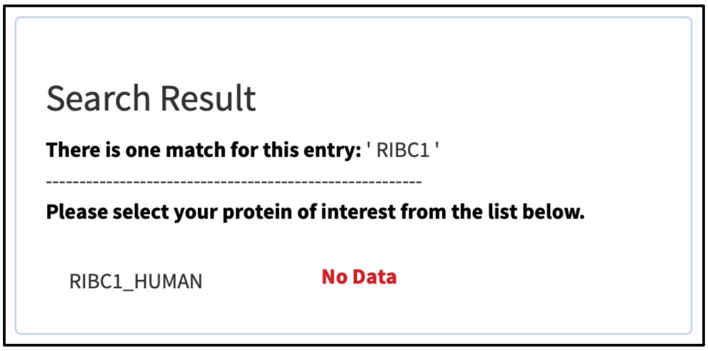

Illustration 8. Example of protein that is not available in the mass spectrometry data.

If there are more than 100 proteins found, the search returns the results for the first 100 matches. Alternatively, the search needs to be refined (**Illustration 9**).

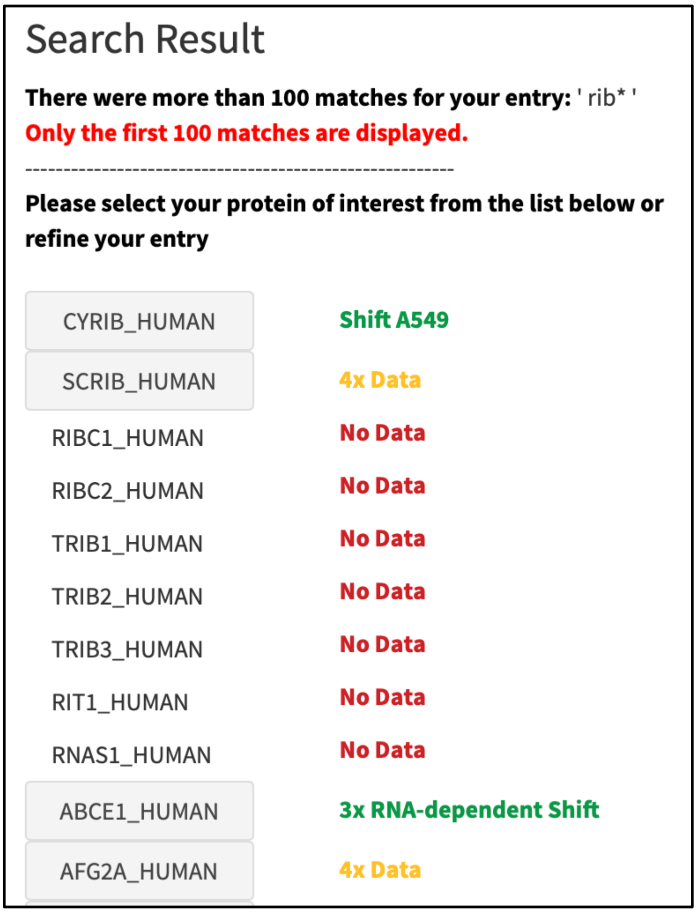

Illustration 9. Search result for more than 100 matches.

By clicking on a specific protein (button), the complete R-DeeP 3.0 analysis results (**Illustration 10**) appear in a new panel, which is composed of several elements.

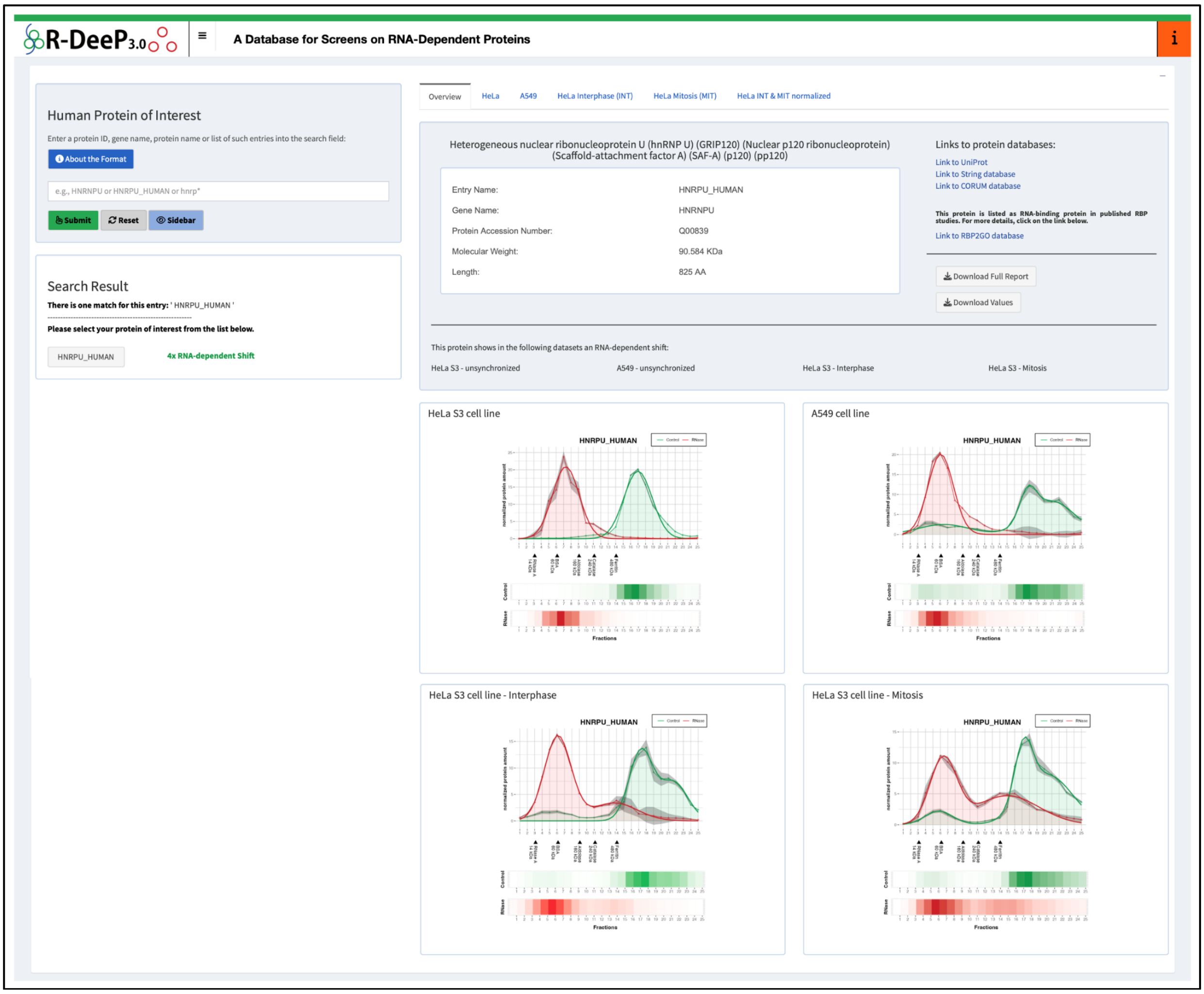

Illustration 10. R-DeeP 3.0 Advanced search analysis results for hnRNP U. On the left side, the "Search Result" panel indicates that the protein depicts four RNA-dependent shifts (*i.e.*, in all four datasets). On the right side, details on the protein are given in various tabs.

For each protein in the "Advanced search", the following information is depicted, if available (additional panels):

- **Overview**: general information about the protein and if available, graphical representation for each dataset. Downloads options are offered.
- **For each additional tab (HeLa, A549, HeLa Interphase (INT)**, **HeLa Mitosis (MIT)**, **HeLa INT & MIT normalized)**: On the left-hand side, the top panel provides the graphical representation of the distribution of the protein in the gradients loaded with control (in green) and RNase-treated (in red) lysates. In the line graph, the respective green and red thin lines are the mean raw data curves for three replicates and the stronger lines are the Gaussian fits of the mean raw data. The grey areas around the curves represent the standard deviation for the raw data calculated from the three replicates. The graph also indicates (black arrows) the position of the standard proteins that were used to calibrate the gradient for the HeLa S3 cell line (**Illustration 11**). Three buttons at the top provide various download options:

- "Download Plot": a PDF of the graphical representation of the distribution of the protein

- “Download Values”: a comma-separated values (CSV) file of the raw data and standard deviation for each fraction
- Download “Full Report”: a complete analysis report in html format

The user can open a larger view of the graphical representation by clicking the “Zoom” button (top left corner of the graph panel).

On the lower left-hand side, the results of the statistical analysis are displayed. Lists of the maxima as well as their position and corresponding amount of protein for the control and RNase gradients are shown. If significant shifts were detected, the parameters of the shifts are indicated.

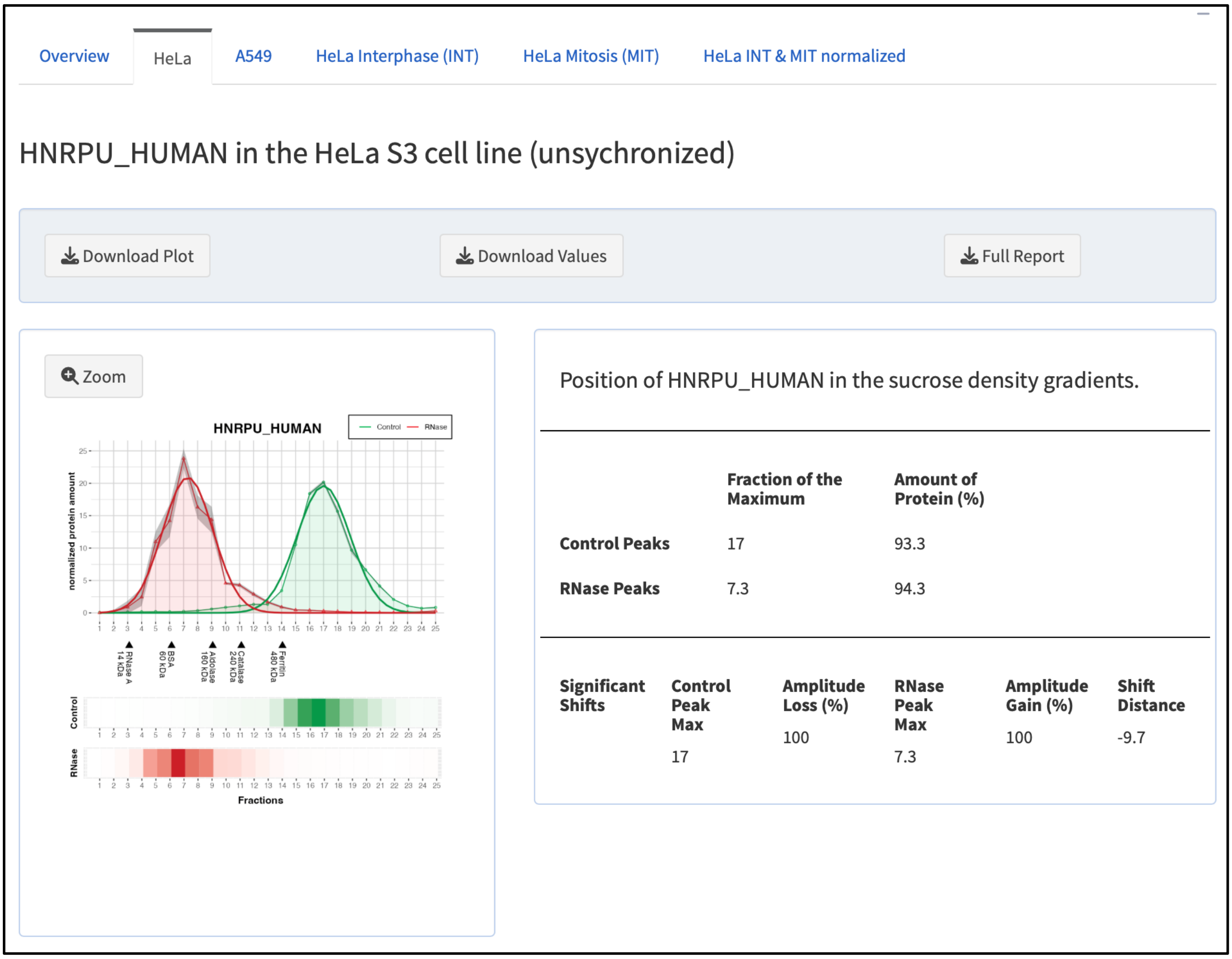

Illustration 11. R-DeeP 3.0 results for hnRNP U in HeLa S3 cells.

2. Single search

This search option allows searching the database for one protein of interest in either of the datasets: HeLa S3 or A549 unsynchronized, HeLa S3 synchronized in Interphase or Mitosis (**Illustration 12**). Once the dataset is selected, it is possible to obtain information for a protein of interest by using the input field (**Illustration 13**).

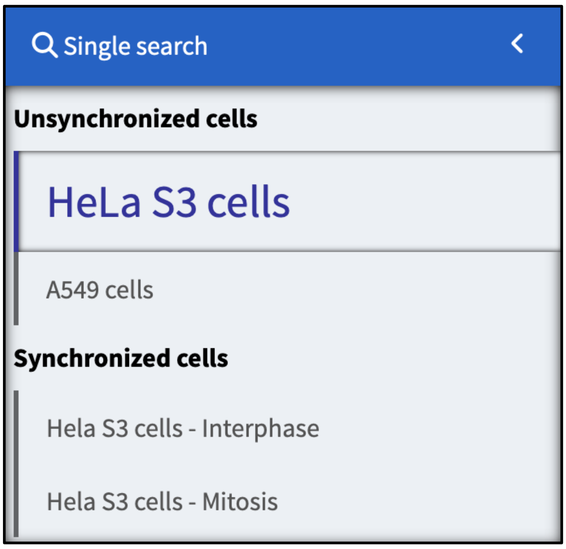

Illustration 12. Dataset selection.

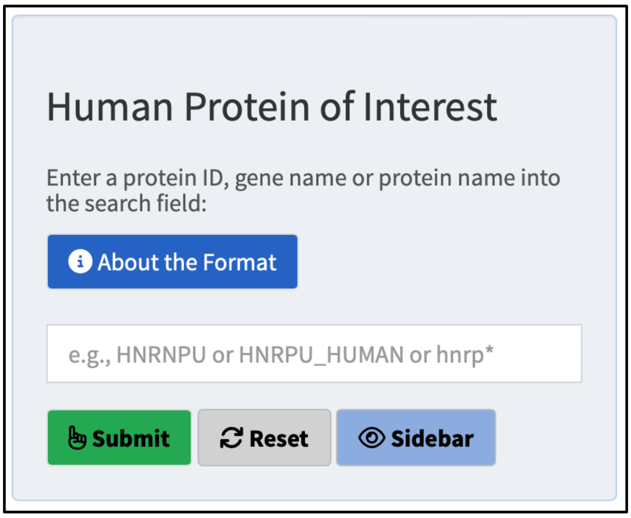

Illustration 13. Single protein search input field.

In the "About the Format" pop-up window, more detailed information about the input format is provided. (**Illustration 14**). The pop-up window contains a link to the UniProt database, that could be helpful to find the proper protein or gene name in case they are not known.

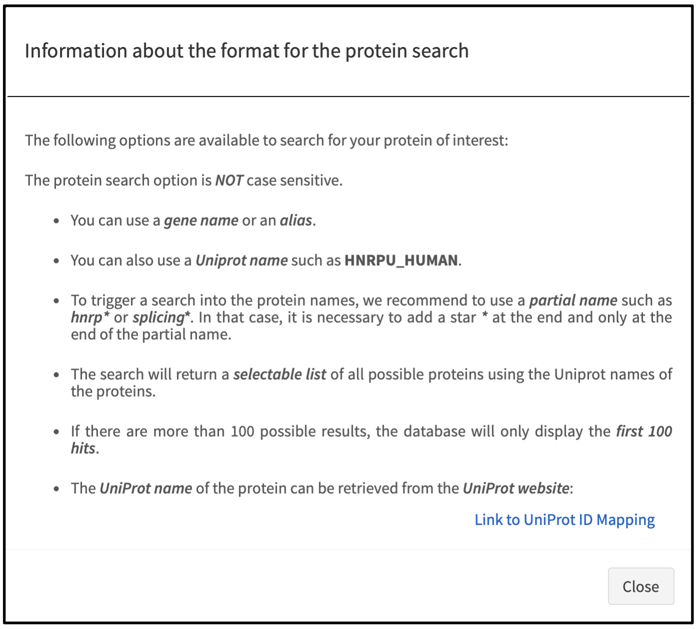

Illustration 14. Information about the search format.

In case of a successful search, a new panel with search results appears with either a unique result (**Illustration 15**) or a list of suggested hits (**Illustration 16**). In case of more than 100 hits, the list contains the first 100 hits (**Illustration 16**). The search result already indicates whether data is available for this protein and whether the protein is shifting (green comment next to the protein name, clickable button), whether there is data available but the protein does not shift (yellow comment, clickable button) or whether there is no data available, *i.e.* the protein has not been

detected by mass spectrometry (red comment, no clickable button, **Illustration 17**). The protein of interest, if available, can be selected to obtain the corresponding comprehensive R-DeeP 3.0 analysis.

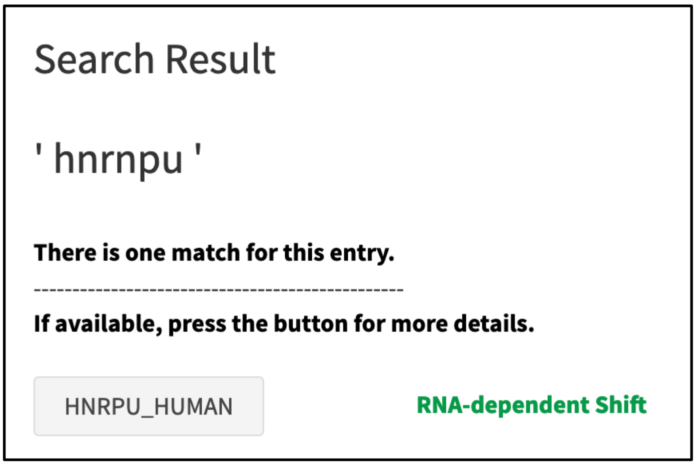

Illustration 15. Result of a search with one match.

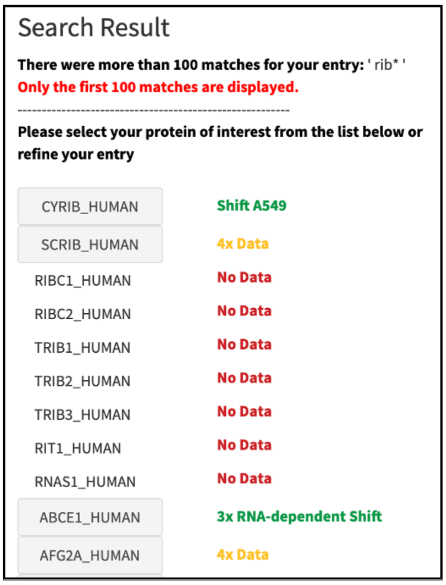

Illustration 16. Result list with multiple hits including message indicating a restriction to the first 100 hits.

The search returns an error message in case there are no results (**Illustration 18**).

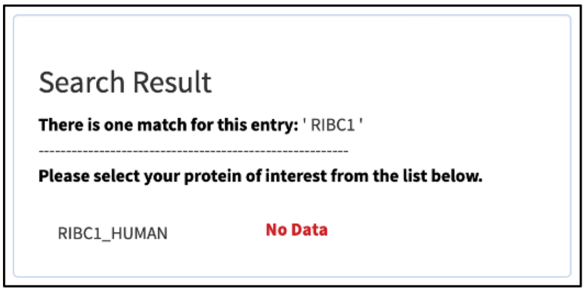

Illustration 17. Example of a protein that is not available in the mass spectrometry data.

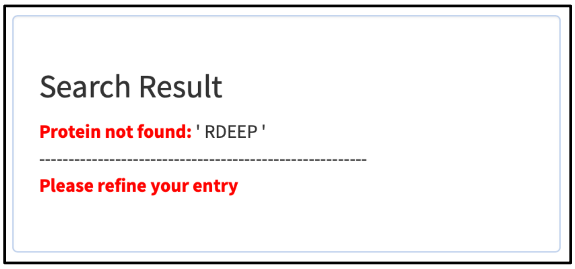

Illustration 18. Error message if no hits are found.

The complete R-DeeP 3.0 analysis results (**Illustration 19**) appear in a new panel, which is composed of several elements.

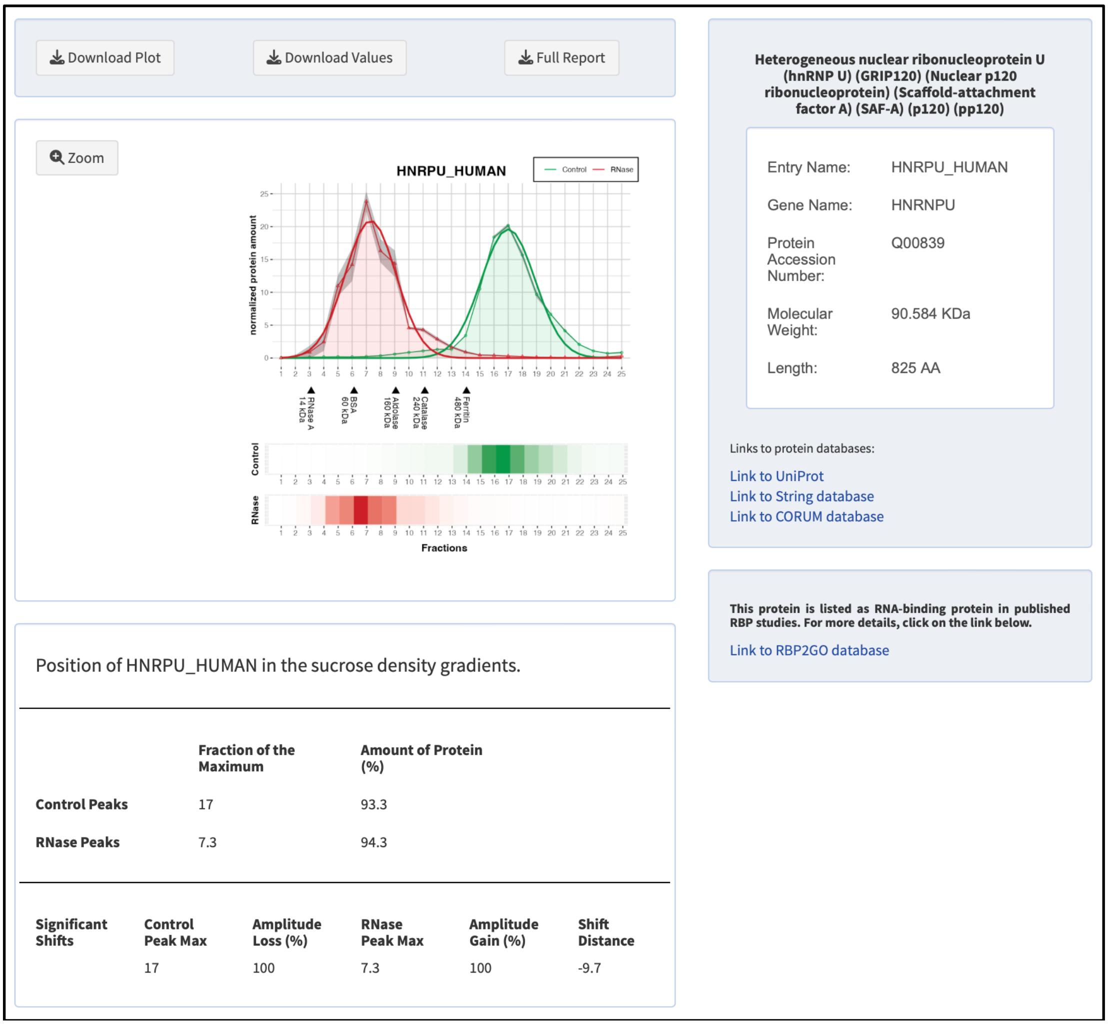

Illustration 19. R-DeeP 3.0 analysis results for hnRNP U.

On the left-hand side, the top panel provides the graphical representation of the distribution of the protein in the gradients loaded with control (in green) and RNase-treated (in red) lysates. In the line graph, the respective green and red thin lines are the mean raw data curves for three replicates and the stronger lines are the Gaussian fits of the mean raw data. The grey areas around the curves represent the standard deviation for the raw data calculated from the three replicates. The graph also indicates (black arrows) the position of the standard proteins that were used to calibrate the gradient for the HeLa S3 cell line.

Three buttons at the top provide various download options:

- “Download Plot”: a PDF of the graphical representation of the distribution of the protein
- “Download Values”: a comma-separated values (CSV) file of the raw data and standard deviation for each fraction
- Download “Full Report”: a complete analysis report in html format

The user can open a larger view of the graphical representation by clicking the “Zoom” button (top left corner of the graph panel).

On the lower left-hand side, the results of the statistical analysis are displayed. Lists of the maxima as well as their position and corresponding amount of protein for the control and RNase gradients are shown. If significant shifts were detected, the parameters of the shifts are indicated.

On the right-hand side, a panel provides information about the protein itself. The top panel offers basic information on the protein as found on the UniProt database as well as direct links to the UniProt page of the protein and the CORUM and STRING database for further information about this protein and its interaction partners (**Illustration 20**). In addition, the lower panel indicates whether the protein of interest has already been listed as potential RNA-binding protein (RBP) in previous studies. A link brings the user directly to the RBP2GO database [4][5] in a new tab.

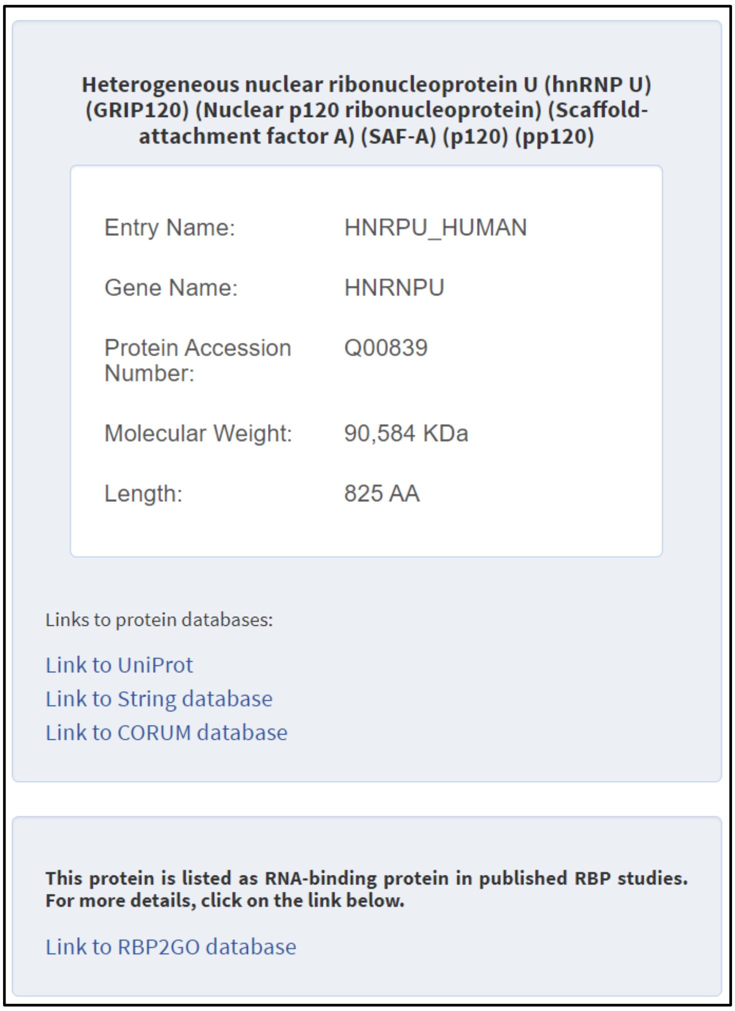

Illustration 20. Panel with specific information on the protein and link to protein databases as well as R-DeeP 3.0 tab for RBP resources.

For the HeLa S3 datasets synchronized in interphase or mitosis, the search results offer the possibility to visualize both results in separate panels at the same time if the protein is available in both datasets (**Illustration 21**). For this visualization, the protein amounts in the graphs were normalized to the cycle-phase for which the protein had the highest amount (**Illustration 22**).

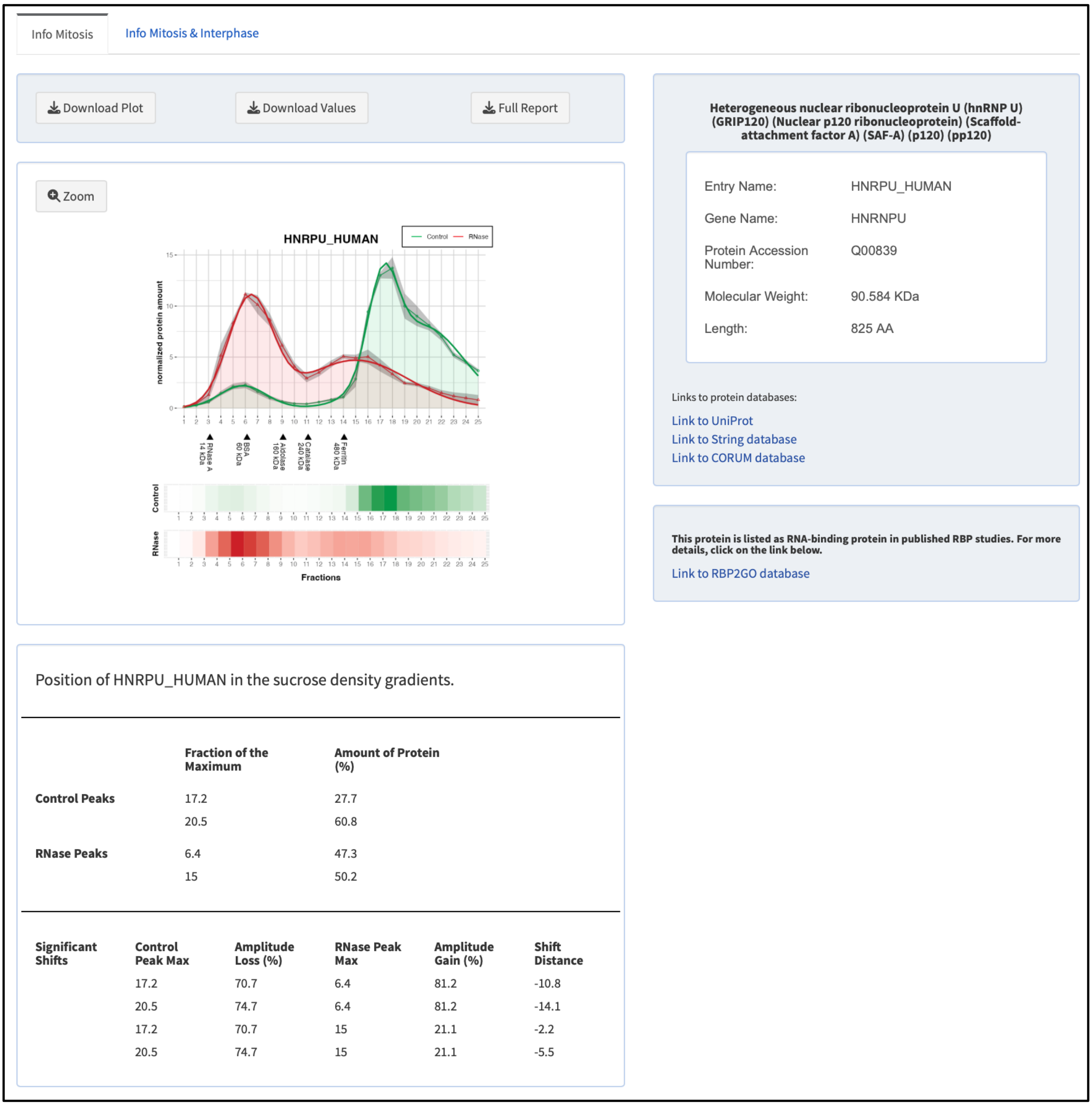

Illustration 21. Panel with specific information on the protein and link to protein databases as well as R-DeeP 3.0 tab for RBP resources.

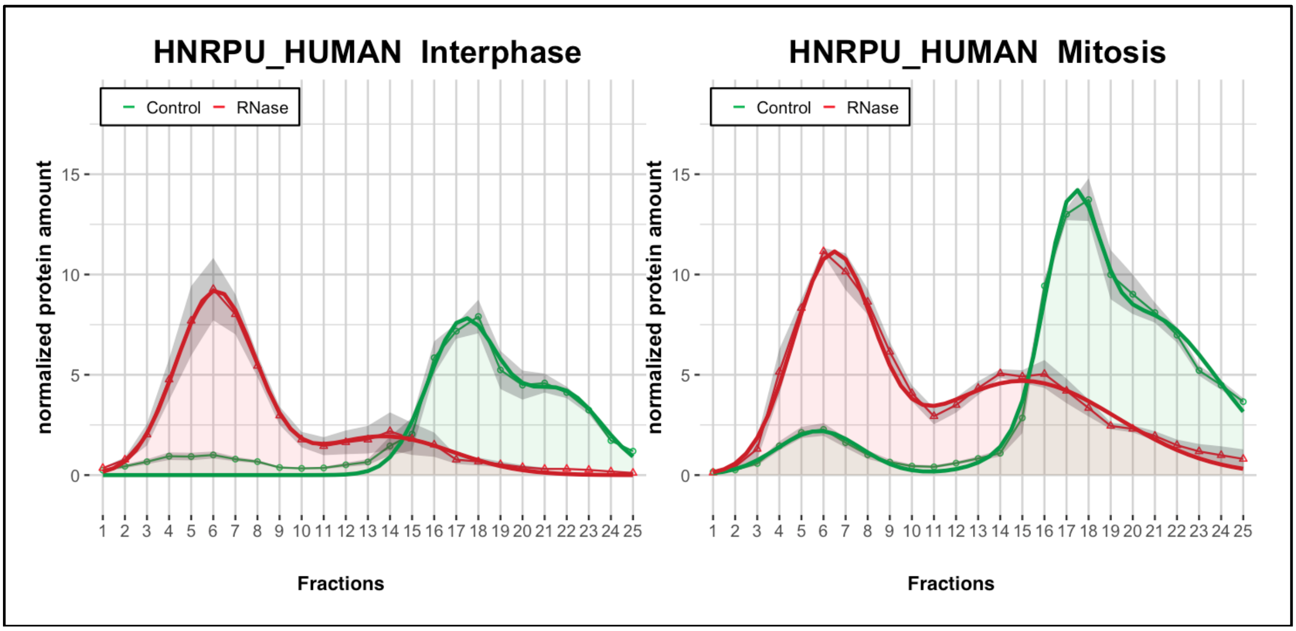

Illustration 22. Graphical representation of the normalized protein amounts in HeLa S3 interphase and mitosis.

3. Other tabs: Links / Documentation / About

The remaining three tabs on the R-DeeP 3.0 homepage (**Illustration 23**) summarize important links, the documentation including this User Guide as well as general information about the R-DeeP 3.0 database including reference to the publication and contact information.

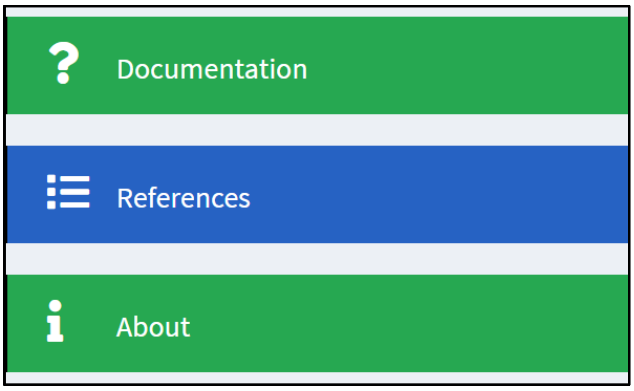

Illustration 23. Documentation, References and About tabs.

4. Example of an R-DeeP 3.0 full report for hnRNP U

R-DeeP 3.0 Analysis report for interphase or mitosis synchronized HeLa S3 cell line

Research Group "RNA-Protein Complexes & Cell Proliferation"

German Cancer Research Center (DKFZ) - Heidelberg - Germany

July 22, 2024

http://r-deep3.dkfz.de
https://rbp2go-2-beta.dkfz.de

References

Caudron-Herger *et al.* 2019, Molecular Cell
Caudron-Herger *et al.* 2020, Nature Protocols
Caudron-Herger *et al.* 2021, Nucleic Acids Research
Rajagopal *et al.* Cancers 2022
Wassmer *et al.* Nucleic Acids Research 2024

UniProt name of the protein: HNRPU_HUMAN

Gene Name: HNRNPU
Gene Name Synonyms: HNRNPU C1orf199 HNRPU SAFA U21.1
Protein Accession Number: Q00839
Molecular Weight: 90.584 KDa
Length: 825 AA
Description: Heterogeneous nuclear ribonucleoprotein U (hnRNP U) (GRIP120) (Nuclear p120 ribonucleoprotein) (Scaffold-attachment factor A) (SAF-A) (p120) (pp120)

RNA-Interaction of the protein: HNRPU_HUMAN

This protein is listed as RNA-binding protein in published RBP studies. For more details, click on the link below.
https://rbp2go-2-beta.dkfz.de

This protein shows in the following R-DeeP 3.0 datasets an RNA-dependent shift:

HeLa S3 (unsynchronized), A549 (unsynchronized), HeLa S3 - Interphase, HeLa S3 - Mitosis

Position of the protein in the sucrose density gradient - HeLa S3 (unsynchronized)

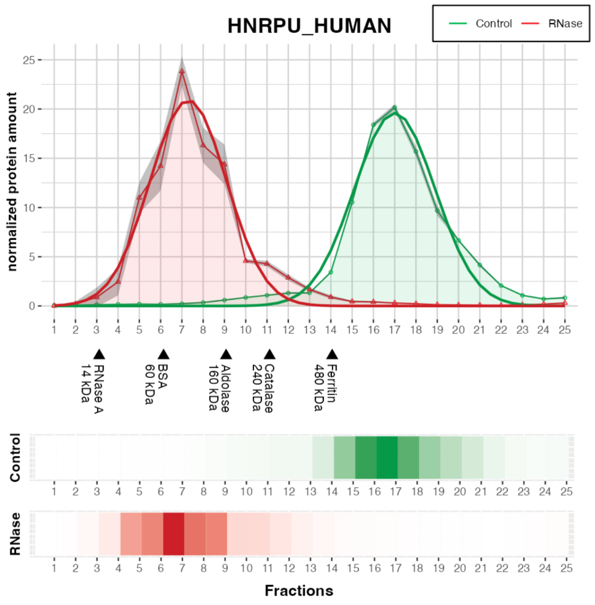

Peaks and shifts information - HeLa S3 (unsynchronized)

Control Peaks

Fraction of the Maximum	Amount of Protein (%)
17	93.3

RNase Peaks

Fraction of the Maximum	Amount of Protein (%)
7.3	94.3

Significant Shifts

Control Peak Max	Amplitude Loss (%)	RNase Peak Max	Amplitude Gain (%)	Shift Distance
17	100	7.3	100	-9.7

Position of the protein in the sucrose density gradient - A549 (unsynchronized)

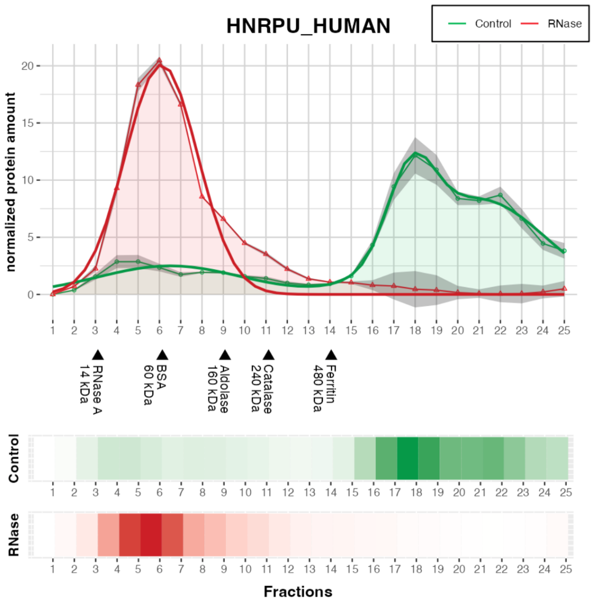

Peaks and shifts information - A549 (unsynchronized)

Control Peaks

Fraction of the Maximum	Amount of Protein (%)
6.5	20.2
17.8	20.1
21.0	58.5

RNase Peaks

Fraction of the Maximum	Amount of Protein (%)
6.1	85.6

Significant Shifts

Control Peak Max	Amplitude Loss (%)	RNase Peak Max	Amplitude Gain (%)	Shift Distance
17.8	100	6.1	87.6	-11.7
21.0	100	6.1	87.6	-14.9

Position of the protein in the sucrose density gradient - HeLa S3 - Interphase

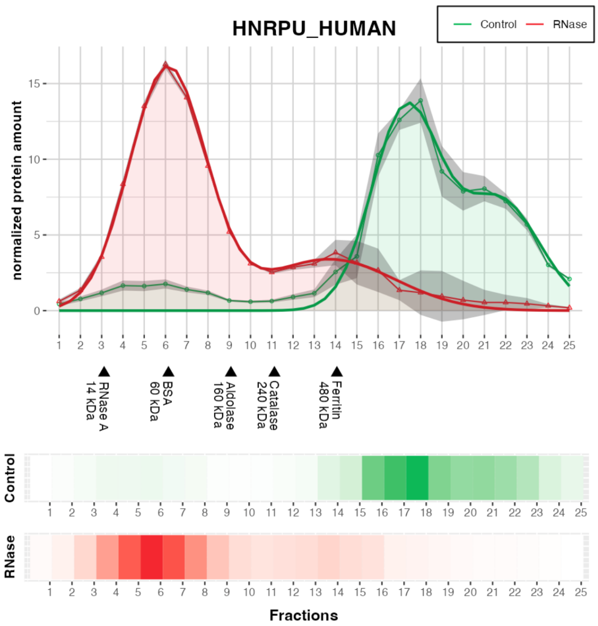

Peaks and shifts information - HeLa S3 - Interphase

Control Peaks

Fraction of the Maximum	Amount of Protein (%)
17.3	52.9
21.7	33.5

RNase Peaks

Fraction of the Maximum	Amount of Protein (%)
6.1	72.1

Significant Shifts

Control Peak Max	Amplitude Loss (%)	RNase Peak Max	Amplitude Gain (%)	Shift Distance
17.3	87.4	6.1	100	-11.2
21.7	98.4	6.1	100	-15.6

Position of the protein in the sucrose density gradient - HeLa S3 - Mitosis

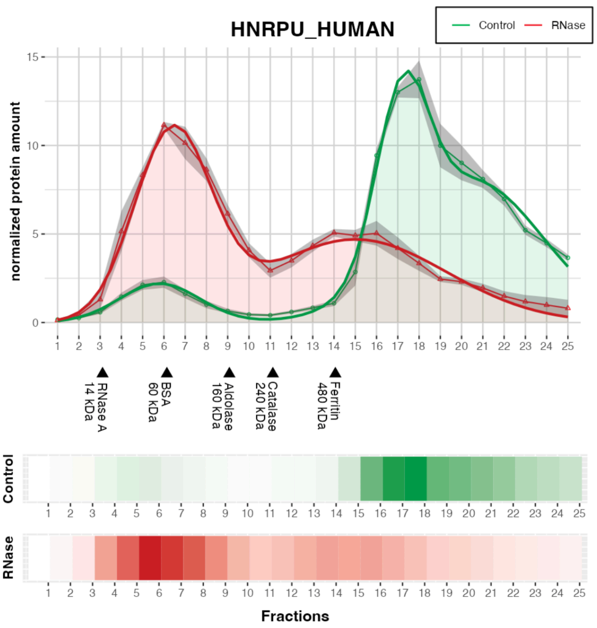

Peaks and shifts information - HeLa S3 - Mitosis

Control Peaks

Fraction of the Maximum	Amount of Protein (%)
17.2	27.7
20.5	60.8

RNase Peaks

Fraction of the Maximum	Amount of Protein (%)
6.4	47.3
15.0	50.2

Significant Shifts

Control Peak Max	Amplitude Loss (%)	RNase Peak Max	Amplitude Gain (%)	Shift Distance
17.2	70.7	6.4	81.2	-10.8
20.5	74.7	6.4	81.2	-14.1
17.2	70.7	15.0	21.1	-2.2
20.5	74.7	15.0	21.1	-5.5

5. Contact information

The authors of the R-DeeP 3.0 database can be contacted at the following email address:

database.rdeep@dkfz.de

6. References

[1] Caudron-Herger, M., Rusin, S.F., Adamo, M.E., Seiler, J., Schmid, V.K., Barreau, E., Kettenbach, A.N. and Diederichs, S. (2019) R-DeeP: Proteome-wide and Quantitative Identification of RNA-Dependent Proteins by Density Gradient Ultracentrifugation. *Mol Cell*, **75**, 184-199 e110.

[2] Rajagopal, V., Loubal, A.S., Engel, N., Wassmer, E., Seiler, J., Schilling, O., Caudron-Herger, M. and Diederichs, S. (2022) Proteome-Wide Identification of RNA-Dependent Proteins in Lung Cancer Cells. *Cancers (Basel)*, **14**, 6109.

[3] Caudron-Herger, M., Wassmer, E., Nasa, I., Schultz, A.S., Seiler, J., Kettenbach, A.N. and Diederichs, S. (2020) Identification, quantification and bioinformatic analysis of RNA-dependent proteins by RNase treatment and density gradient ultracentrifugation using R-DeeP. *Nat Protoc*, **15**, 1338-1370.

[4] Caudron-Herger, M., Jansen, R.E., Wassmer, E. and Diederichs, S. (2021) RBP2GO: a comprehensive pan-species database on RNA-binding proteins, their interactions and functions. *Nucleic Acids Res*, **49**, D425-D436.

[5] Wassmer, E., Koppany, G., Hermes, M., Diederichs, S. and Caudron-Herger, M. (2024) Refining the pool of RNA-binding domains advances the classification and prediction of RNA-binding proteins. *Nucleic Acids Res*, **52**, 7504–7522.
