# Supplementary material for: An atlas of RNA-dependent proteins in cell division reveals the riboregulation of mitotic protein-protein interactions
Source: Nat Commun. 2025 Mar 8;16:2325. doi: 10.1038/s41467-025-57671-3 (PMC11890761; doi:10.1038/s41467-025-57671-3)
Supplement: Supplementary file 2 — Description of Additional Supplementary Files [file 41467_2025_57671_MOESM2_ESM.pdf]

Description of Additional Supplementary Files

Supplementary Data 1: Results of the cell cycle-related R-DeeP approach in mitosis and interphase

Supplementary Data 2: Atlas of RNA-dependent proteins in cell division

Supplementary Data 3: Analysis of the AURKA interaction partners

Supplementary Data 4: iCLIP2-Seq analysis of KIFC1-interacting RNAs

Supplementary Data 5: List of reagents
