# Supplementary material for: An atlas of RNA-dependent proteins in cell division reveals the riboregulation of mitotic protein-protein interactions
Source: Nat Commun. 2025 Mar 8;16:2325. doi: 10.1038/s41467-025-57671-3 (PMC11890761; doi:10.1038/s41467-025-57671-3)
Supplement: Supplementary file 7 — Supplementary Data 5 [file 41467_2025_57671_MOESM7_ESM.pdf]

Supplementary Table 5: List of reagents

Experimental models & Cell lines	Catalogue number	Company
HeLa	CCL-2	ATCC
A549	CCL-185	ATCC

Reagents or Resources	Catalogue number	Lot number	Company
DMEM High Glucose medium	D65796	N/A	Sigma-Aldrich
RPMI 1640 medium	11875093	N/A	Gibco, Thermo Fisher Scientific
Dulbecco's Phosphate Buffered Saline (PBS)	D8537	N/A	Sigma-Aldrich
Luria Broth Base (LB)	12795-027	200806062408	Invitrogen
Human KIFC1 ORF	HG15958-G	N/A	Sino Biologicals
NEB 5-alpha Competent E. coli (High Efficiency)	C2987H	N/A	New England Biolabs (NEB)
One Shot TOP10 Chemically Competent cells E. coli	C404003	N/A	Thermo Fisher Scientific
Q5 Site-Directed Mutagenesis Kit	E0554S	N/A	New England Biolabs (NEB)
E. coli Ribonuclease H (RNase H), 2 U/µl	8021014		Thermo Fisher Scientific
RNase A	10109169001	49574222	Roche Diagnostics GmbH
Nucleospin plasmid isolation kit	740588.250	1707/002	Macherey Nagel
GeneJET gel extraction kit	K0692	00439790	Thermo Scientific
RNA clean and concentrator-5	R1014	209311	Zymo Research
Gateway™ BP Clonase™ II Enzyme-Mix	11789100	N/A	Thermo Fisher Scientific
Gateway TM LR Clonase TM II Enzyme Mix	11791020	N/A	Thermo Fisher Scientific
Proteinase K	AM2546		Ambion
2x Phusion High-Fidelity PCR MasterMix	M0531S	N/A	New England Biolabs (NEB)
Tissue culture dish 146 x 21 mm (15 cm)	93150	N/A	Techno Plastic Products
Thymidine cell culture tested	T1895-1G	N/A	Sigma-Aldrich
Methyl-(5-(2-thienylcarbonyl)-1H-*benzim (Nocodazole)	M1404-2MG	N/A	Sigma-Aldrich

4–20% Criterion™ TGX Stain-Free™ Protein Gel, 26 well, 15 µl	5678095	N/A	BioRad
7.5% precast Mini-Protean-TGX gel, 10 wells	456-1024	N/A	BioRad
7.5% precast Mini-Protean-TGX gel, 12 wells	456-1025	N/A	BioRad
7.5% precast Mini-Protean-TGX gel, 15 wells	456-1026	N/A	BioRad
NuPAGE LDS Sample Buffer	NP0007	2538211	Thermo Fisher Scientific
Page Ruler Prestained Protein Ladder	26616	N/A	Thermo Fisher Scientific
Pager Ruler Plus Prestained Protein Ladder	26619	N/A	Thermo Fisher Scientific
Bovine Serum Albumin (BSA)	A1470	10042749	Sigma-Aldrich
Bicinchoninic Acid solution	B9643-1L	N/A	Sigma-Aldrich
Pierce™ BCA Protein Assay Reagent B	23224	TA260103	Thermo Fisher Scientific
Amersham™ Protran® Western blotting membranes, nitrocellulose	10600002	N/A	Sigma-Aldrich
5x Trans-Blot Turbo Transfer Buffer	10026938	N/A	Bio-Rad
HRP-conjugated Goat anti-mouse secondary antibody	115-035-003	108284	Dianova
HRP-conjugated Goat anti-rabbit secondary antibody	111-035-144	149084	Dianova
ECL prime Western blotting system	RPN2232	N/A	Cytiva
Proteus clarification mini spin column	42225.01	N/A	Serva
Pierce ChIP-grade Protein A/G Magnetic Beads	26162	XH353638	Thermo Fisher Scientific
Dynabeads Protein A	10002D	01268693	Thermo Fisher Scientific
Dynabeads Protein G	10004D	01265132	Thermo Fisher Scientific
DynaMagTM-2 magnet	12321D	N/A	Thermo Fisher Scientific
GFP-Trap Magnetic Agarose beads	gtma-20	208182-02-02	Chromotek
NegC.<br>5nmol		n.A.	siPOOL AURKa-c1 NCBI Gene ID:6790; 5nmol
siPOOL AURKa-c1 NCBI Gene ID:6790; 5nmol		AURKA-c1-002	siTOOLs Biotech

RNAiMAX Transfection Reagent	13778150	3011788	Life Technologies
Complete, EDTA-free Protease Inhibitor Cocktail	4693132	73791000	Sigma-Aldrich
PhosStop	4906845001	73124700	Sigma-Aldrich
TURBO DNase	AM2238	01341010	Thermo Fisher Scientific
RNase I 100 U/µl	AM2295	01317147	Thermo Fisher Scientific
Normal rabbit IgG	12-370	N/A	Millipore
Aurora A (D3E4Q) Rabbit monoclonal antibody	14475	Lot2	Cell Signaling Technology
Anti-AURKA (1F8), Mouse monoclonal antibody	12100	Lot1	Cell Signaling Technology
KIFC1 antibody (11445), Rabbit monoclonal antibody	172620	GR3259790-3	Abcam
Anti-Histone H3 (phospho S10) antibody	ab5176	GR3242359-1	Abcam
ß-Actin antibody	A2228	067M4856V	Sigma-Aldrich
Anti-TPX2, Clone TPX2-01	SAB4701065	539147	Sigma-Aldrich
Anti-TPX2 (18D5)	628002	B256728	Biolegend
Anti GAPDH	MAB374	3169854	Millipore
Cell culture plate, 12-well	92412	N/A	Techno Plastic Products
Microscope cover glasses, 12 mm, Nr. 1.5	0112520	51687	Neolab
Duolink® In Situ Detection Reagents Red	DUO92008	0000326527	Sigma-Aldrich
Duolink® In Situ Wash Buffers, Fluorescence	DUO82049	N/A	Sigma-Aldrich
Duolink® In Situ PLA® Probe Anti-Mouse MINUS, Affinity purified Donkey anti-Mouse IgG (H+L)	DUO92004	0000337233	Sigma-Aldrich
Duolink® In Situ PLA® Probe Anti-Rabbit PLUS, Affinity purified Donkey anti-Rabbit IgG (H+L)	DUO92002	SLCR1898	Sigma-Aldrich
Duolink In Situ Mounting medium	DUO82040-5ML	0000339465	Sigma-Aldrich
Alexa Fluor 488 goat anti-rabbit IgG	A11034	VG302077	Thermo Fisher Scientific
Alexa Fluor 488 goat anti-mouse IgG	A32723	VH309036	Thermo Fisher Scientific
T4 PNK 10U/µl 500 µl	M0201	10129472	New England Biolabs (NEB)

ATP 10 mM 1 ml	P0756	10109057	New England Biolabs (NEB)
[gamma-P32] Adenosine 5'-triphosphate (ATP) /9,25 MBq	SRP-501		Hartmann Analytic
SUPERase-In RNase Inhibitor (20 U/µL)-10,000 units	AM2696		Thermo Fisher Scientific
Phenol-chloroform-Isoamylalkohol pH 6.5-6.9	P3803		Sigma-Aldrich
Heavy Phase Lock Gel tubes	733-2478		SERVA
T4 RNA Ligase 1 (ssRNA Ligase), High Concentration	M0437M		NEB
Superscript IV reverse transcriptase	18090050		Thermo Fisher Scientific
MyONE silane beads	37002D		Thermo Fisher Scientific
RLT Buffer	79216		Qiagen
RNaseOUT, 40 U/µl	10777-019		
ProNex® Size-Selective Purification System	NG2001		Promega
RNA Clean & Concentrator™-5 (50 Preps) w/ Zymo-Spin™ IC Columns (Capped)	R1015		Zymo Research
Ribolock RNase Inhibitor (40 U/µL)	EO0384	00965329	Thermo Fisher Scientific
Lipofectamine 2000 Transfection Reagent	11668500		Thermo Fisher Scientific
Opti-MEM(R) I Reduced Serum Medium	31985047		Thermo Fisher Scientific
Pierce™ Anti-DYKDDDDK Magnetic Agarose	A36797	YD368599	Thermo Scientific
Human AURKA protein	N/A	N/A	EMBL Protein Expression and Purification Core Facility
Human AURKA-D274A protein	N/A	N/A	EMBL Protein Expression and Purification Core Facility
Human KIFC1 protein	N/A	N/A	EMBL Protein Expression and Purification Core Facility
Human TPX2 protein	N/A	N/A	EMBL Protein Expression and Purification Core Facility

**Others**	**Catalogue Number**	**Company**

Imager ECL Chemo Cam CC5569	N/A	INTAS
Stratalinker 2400	N/A	Stratagene
SW 40 Ti Swinging-Bucket Rotor	331302	Beckman Coulter
Ultra-Clear Tube	344060	Beckman Coulter
Orbitrap Fusion LC-MS/MS platform	N/A	Thermo Fisher Scientific
Fusion Orbitrap Lumos mass spectrometer	N/A	Thermo Fisher Scientific
Discovery 90SE Ultracentrifuge	N/A	Sorvall
Prometheus NT.48 nanoDSF	N/A	NanoTemper Technologies GmbH
Sequencing Grade Modified Trypsin	V5113	Promega
TMTsixplex™ Isobaric Label Reagent Set	90066	Thermo Fisher Scientific

**Softwares**		
Bioconductor	Gentleman et al., 2004	www.bioconductor.org
Comet	Eng et al., 2013	http://comet-ms.sourceforge.net
ImageJ	Schneider et al., 2012	https://imagej.net/Downloads
LabImage 1D 2006	Kapelan Bio-Imaging GmbH	www.labimage.com
Microsoft Excel	Microsoft	www.microsoft.com
Primer Blast	NCBI	https://www.ncbi.nlm.nih.gov/tools/primer-blast/
R programming	The R project	www.r-project.org
Shiny	R Studio	https://shiny.rstudio.com
GO Enrichment Analysis	Thomas PD et al., 2022	https://geneontology.org/
Serial cloner	Serial Basics	http://serialbasics.free.fr/Serial_Cloner.html
GIMP	The GNU Project	https://www.gimp.org
Ensemble	Harrison et al., 2024	https://www.ensembl.org/index.htm

Primers and other sequences			
**Cloning primers**	**Sequence**		**Company**
KIFC1 Forward	GGGGACAAGTTTGTACAAAAAAGCAGGCTTCATGGATCCGCAGAGGTCCCCCTATTG	Primers to generate Entry clone for Gateway cloning.	Sigma-Aldrich
KIFC1 Reverse	GGGGACCACTTTGTACAAGAAAGCTGGGTTCTATCACTTCCTGTTGGCCTGAGCAGTACC		Sigma-Aldrich
S6A_FP	TCCGCAGAGGGCCCCCTATTGG		Sigma-Aldrich
S6A_RP	TCCATTCACTTCCTGTTGGCCTGAGC		Sigma-Aldrich
S26A_FP	TAAGGCCCCTGCCCAGCTGCCTC		Sigma-Aldrich
S26A_RP	ATCAGAGGTCTCTTCAGTTCTATGTTCCCCTTTAC		Sigma-Aldrich
S31A_FP	GCTGCCTCTCGCAGGAAGCAGAC		Sigma-Aldrich
S31A_RP	TGGGAAGGGGCCTTAATCAG		Sigma-Aldrich
S349A_FP	AACCCGCCTTGCGCTCTCCCGGTCTGAC		Sigma-Aldrich
S349A_RP	GGAGGATCAGAGGGCCCA		Sigma-Aldrich
T359A_FP	GCGGCGTGGGGCCCTGAGTGGGG		Sigma-Aldrich
T359A_RP	TCGTCAGACCGGGAGAGGCTAAGGCG		Sigma-Aldrich
pFastBac_KIFC1-FP	ATGGATCCGGAATTCAAAG	Primer for NEBuilder Cloning to generate KIFC1 for expression in insect cells	Sigma-Aldrich
pFastBac_KIFC1-RP	GGCGCCCTGAAAATACAG		Sigma-Aldrich
KIFC1-FP	ACCTGTATTTTCAGGGCGCCATGGATCCGCAGAGGTCC		Sigma-Aldrich
KIFC1-RP	CCTTTGAATTCCGGATCCATTCACTTCCTGTTGGCCTG		Sigma-Aldrich
pFastBac_AURKA-FP	ACAGTCTTAGTAATCAGCCATACCACATTTG	Primer for NEBuilder Cloning to generate AURKA for expression in insect cells	Sigma-Aldrich
pFastBac_AURKA-RP	ATCGGTCCATGGCGCCCTGAAAATACAG		Sigma-Aldrich
AURKA-FP	TCAGGGCGCCATGGACCGATCTAAAGAAAAC		Sigma-Aldrich
AURKA-RP	TGGCTGATTACTAAGACTGTTTGCTAGC		Sigma-Aldrich
pFastBac_TPX2-FP	CCACTGCTAATAATCAGCCATACCACATTTG	Primer for NEBuilder Cloning to generate AURKA for expression in insect cells	Sigma-Aldrich
pFastBac_TPX2-RP	CTTGTGACATGGCGCCCTGAAAATACAG		Sigma-Aldrich
TPX2-FP	TCAGGGCGCCATGTCACAAGTTAAAAGCTC		Sigma-Aldrich
TPX2-RP	TGGCTGATTATTAGCAGTGGAATCGAGTG		Sigma-Aldrich
D274A_FP	TAAAATTGCAGCTTTTGGGTGGTC		Sigma-Aldrich

D274A_RP	AGCTCTCCAGCTGATCCA		Sigma-Aldrich
**iCLIP2 primers, adapters or barcode sequences**			
**Adapters or barcodes or primers**	**Sequence**		**Company**
L3-App	/5rApp/AG ATC GGA AGA GCG GTT CAG /3ddC/		IDT
RT oligo	GGATCCTGAACCGCT		Sigma-Aldrich
P3Solexa_s	CACGACGCTCTTCCGATCT		Sigma-Aldrich
P5Solexa_s	CTGAACCGCTCTTCCGATCT		Sigma-Aldrich
P3Solexa	AATGATACGGCGACCACCGAGATCTACACT CTTTCCCTACACGACGCTCTTCCGATCT		Sigma-Aldrich
P5Solexa	CAAGCAGAAGACGGCATACGAGATCGGTC TCGGCATTCCTGCTGAACCGCTCTTCCGATC T		Sigma-Aldrich
L02clip2.0	/5Phos/NN NNC GAT GTN NNN NAG ATC GGA AGA GCG TCG TG/3ddC/		IDT
L05clip2.0	/5Phos/NN NNA CAG TGN NNN NAG ATC GGA AGA GCG TCG TG/3ddC/		IDT
L10clip2.0	/5Phos/NN NNT AGC TTN NNN NAG ATC GGA AGA GCG TCG TG/3ddC/		IDT
L19clip2.0	/5Phos/NN NNG TGA AAN NNN NAG ATC GGA AGA GCG TCG TG/3ddC/		IDT
