# Supplementary material for: An atlas of RNA-dependent proteins in cell division reveals the riboregulation of mitotic protein-protein interactions
Source: Nat Commun. 2025 Mar 8;16:2325. doi: 10.1038/s41467-025-57671-3 (PMC11890761; doi:10.1038/s41467-025-57671-3)
Supplement: Supplementary file 8 — Reporting Summary [file 41467_2025_57671_MOESM8_ESM.pdf]

nature portfolio

Corresponding author(s): Maïwen Caudron-Herger

Last updated by author(s): Feb 27, 2025

Reporting Summary

Nature Portfolio wishes to improve the reproducibility of the work that we publish. This form provides structure for consistency and transparency in reporting. For further information on Nature Portfolio policies, see our Editorial Policies and the Editorial Policy Checklist.

Statistics

For all statistical analyses, confirm that the following items are present in the figure legend, table legend, main text, or Methods section.

n/a	Confirmed	
☐	☒	The exact sample size (*n*) for each experimental group/condition, given as a discrete number and unit of measurement
☐	☒	A statement on whether measurements were taken from distinct samples or whether the same sample was measured repeatedly
☐	☒	The statistical test(s) used AND whether they are one- or two-sided<br>*Only common tests should be described solely by name; describe more complex techniques in the Methods section.*
☒	☐	A description of all covariates tested
☐	☒	A description of any assumptions or corrections, such as tests of normality and adjustment for multiple comparisons
☐	☒	A full description of the statistical parameters including central tendency (e.g. means) or other basic estimates (e.g. regression coefficient) AND variation (e.g. standard deviation) or associated estimates of uncertainty (e.g. confidence intervals)
☐	☒	For null hypothesis testing, the test statistic (e.g. *F*, *t*, *r*) with confidence intervals, effect sizes, degrees of freedom and *P* value noted<br>*Give P values as exact values whenever suitable.*
☒	☐	For Bayesian analysis, information on the choice of priors and Markov chain Monte Carlo settings
☒	☐	For hierarchical and complex designs, identification of the appropriate level for tests and full reporting of outcomes
☒	☐	Estimates of effect sizes (e.g. Cohen's *d*, Pearson's *r*), indicating how they were calculated

Our web collection on statistics for biologists contains articles on many of the points above.

Software and code

Policy information about availability of computer code

Data collection	Datasets collected from RBP2GO-2-beta.dkfz.de (database maintained by our group)<br>Gene ontology resource (https://geneontology.org)
Data analysis	- Rstudio version 4.3.2 and RStudio Workbench version 4.3.0 and 4.4.0<br>- R libraries (BiocStyle, rtracklayer, ggplot2, BindingSiteFinder, png, ComplexHeatmap, dplyr, ggpubr, gridExtra, GenomicFeatures, forcats, paletteer, tidyr, GGally, BSgenome.Hsapiens.UCSC.hg38, plyranges, ggpubr, ggseqlogo, msa, ggpointdensity, pqsfinder, RColorBrewer, cliProfiler, cowplot, viridis, circlize, knitr, matrixStats, Gviz, ggrepel, limma, edgeR, ggrepel, pheatmap, viridis, knitr, AnnotationHub, clusterProfiler, shiny, shinydashboard, shinyjs, raster, graphics, grid, gridExtra, lattice, DT, magrittr, rmarkdown, shinycssloaders, formattable, shinybusy)<br>- MaxQuant (using an organism specific database extracted from Uniprot.org (human reference database, containing 74,811 unique entries from 27th February 2020)<br>- ImageJ (version 2.1.0/1.54h)<br>- LabImage 1D 2006 (www.labimage.com)<br>- Primer Blast (https://www.ncbi.nlm.nih.gov/tools/primer-blast/)<br>- Serial cloner (http://serialbasics.free.fr/Serial_Cloner.html)<br>- Imager ECL Chemo Cam CC5569 (INTAS)

For manuscripts utilizing custom algorithms or software that are central to the research but not yet described in published literature, software must be made available to editors and reviewers. We strongly encourage code deposition in a community repository (e.g. GitHub). See the Nature Portfolio guidelines for submitting code & software for further information.

Data

Policy information about availability of data

All manuscripts must include a data availability statement. This statement should provide the following information, where applicable:

- Accession codes, unique identifiers, or web links for publicly available datasets
- A description of any restrictions on data availability
- For clinical datasets or third party data, please ensure that the statement adheres to our policy

The R-DeeP 3.0 database is available online at https://R-DeeP3.dkfz.de.
The accession number for the R-DeeP screen proteomic dataset is PXD056068 at ProteomeXchange.
The accession number for the AURKA interactor analysis proteomic dataset is PXD056233 at ProteomeXchange.
The accession number for the KIFC1 iCLIP datasets (4 replicates) is E-MTAB-14472 at ArrayExpress.
The accession number for the RNA-seq (total RNA, 3 replicates) is E-MTAB-14754 at ArrayExpress.

Research involving human participants, their data, or biological material

Policy information about studies with human participants or human data. See also policy information about sex, gender (identity/presentation), and sexual orientation and race, ethnicity and racism.

Reporting on sex and gender	n/a
Reporting on race, ethnicity, or other socially relevant groupings	n/a
Population characteristics	n/a
Recruitment	n/a
Ethics oversight	n/a

Note that full information on the approval of the study protocol must also be provided in the manuscript.

Field-specific reporting

Please select the one below that is the best fit for your research. If you are not sure, read the appropriate sections before making your selection.

☒ Life sciences ☐ Behavioural & social sciences ☐ Ecological, evolutionary & environmental sciences

For a reference copy of the document with all sections, see nature.com/documents/nr-reporting-summary-flat.pdf

Life sciences study design

All studies must disclose on these points even when the disclosure is negative.

Sample size	- For the R-DeeP screens, cells were grown on 15 cm plates in order to obtain 1 mg of protein lysate per replicate and per sample.<br>- For synchronization, cells amounts were calculated so that the cells were about 80% confluent at prometaphase or metaphase.<br>- For image analysis, 10 cells were analyzed per conditions and per replicate.<br>- For immunoprecipitation followed by MS analysis, measurements were performed in four replicates due to the possible variability of the results.<br>- iCLIP2 analysis was also performed in four replicates.<br>- RNA-seq was performed in three replicates.<br>- Details can be found in the method section.
Data exclusions	Replicate 4 of the AURKA immunoprecipitation analysis followed by MS analysis was excluded from the analysis as it clustedred differently from the other replicates.
Replication	Reproducibility was tested using correlation or clustering analyses.
Randomization	Lysates and cells between replicates were collected on different days and randomly assigned to control and treatment conditions.
Blinding	Mass spectrometry and iCLIP2 samples were blinded for our collaborators and core facilities.

Reporting for specific materials, systems and methods

We require information from authors about some types of materials, experimental systems and methods used in many studies. Here, indicate whether each material, system or method listed is relevant to your study. If you are not sure if a list item applies to your research, read the appropriate section before selecting a response.

Materials & experimental systems

n/a	Involved in the study
☐	☒ Antibodies
☐	☒ Eukaryotic cell lines
☒	☐ Palaeontology and archaeology
☒	☐ Animals and other organisms
☒	☐ Clinical data
☒	☐ Dual use research of concern
☒	☐ Plants

Methods

n/a	Involved in the study
☒	☐ ChIP-seq
☒	☐ Flow cytometry
☒	☐ MRI-based neuroimaging

Antibodies

Antibodies used	- Aurora A (D3E4Q) Rabbit monoclonal antibody (#14475, Cell Signaling Technology)<br>- Anti-AURKA (#12100, Cell Signaling)<br>- KIFC1 antibody (11445), Rb monoclonal antibody (#172620, Abcam)<br>- Anti-TPX2, Clone TPX2-01 (#SAB4701065, Sigma-Aldrich)<br>- Anti-TPX2 (18D5) (#628002, Biolegend)<br>- anti-GAPDH (#MAB374, Millipore)<br>- anti-beta-actin (#A2228, Sigma-Aldrich)<br>- Alexa Fluor 488 goat anti-rabbit IgG (#A11034, Thermo Fisher Scientific)<br>- Alexa Fluor 488 goat anti-mouse IgG (#A32723, Thermo Fisher Scientific)
Validation	Validation of the antibodies was provided by the manufacturers.<br>Specificity of the antibody was verified by western blotting on whole cell lysate (anti AURKA, anti TPX2 and Anti KIFC1).

Eukaryotic cell lines

Policy information about cell lines and Sex and Gender in Research

Cell line source(s)	- HeLa (#CCL-2, ATCC)<br>- A549 (#CCL-185, ATCC)
Authentication	Provided by the manufacturer and in addition, all cell lines were authenticated by gDNA analysis on a regular basis (Microsynth AG, Switzerland).
Mycoplasma contamination	All cell lines were tested negative for mycoplasma contamination (PCR test)
Commonly misidentified lines<br>(See ICLAC register)	n/a

Plants

Seed stocks	n/a
Novel plant genotypes	n/a
Authentication	n/a
